# Analysis of a Novel Human Protein, ORF3, Encoded by Spacer rDNA

**DOI:** 10.1007/s00239-025-10269-1

**Published:** 2025-09-19

**Authors:** Yaohui Chen, Jung-Hyun Kim, Hee-Sheung Lee, Sergey Koren, Vladimir Larionov, Adam M. Phillippy, David Schlessinger, Ramaiah Nagaraja

**Affiliations:** 1 Laboratory of Genetics and Genomics, Biomedical Research Center, National Institute on Aging, National Institutes of Health Intramural Research Program (NIH IRP), 251 Bayview Blvd., Baltimore, MD 21224 USA; 2 Developmental Therapeutics Branch, National Cancer Institute, NIH IRP, Bethesda, MD 20892 USA; 3 Genome Informatics Section, Center for Genomics and Data Science Research, National Human Genome Research Institute, NIH, Bethesda, MD 20892 USA

**Keywords:** Ribosomal RNA, Senescence, Cancer cells

## Abstract

An open reading frame in the intergenic spacer of human ribosomal (r)DNA codes for a 190 amino acid, 22 kDa protein that we have named ORF3. It comprises a 5’AluSx repeat sequence encoding 96 amino acids followed by a stretch of 94 amino acids containing a unique repeated stretch of 5 hydrophobic residues. Full copies of *ORF3* have been isolated as transformation-associated recombination clones from mouse:human hybrid cell lines containing human chromosomes 21 or 22. In initial instances where the chromosome complement of rDNA repeats is fully resolved in whole genomes, in CHM13 cells, complete copies of *ORF3* are mainly concentrated in a tandem cluster on chromosome 21, while other chromosomes contain 1 or 2 full copies, with the sequence in other rDNA repeats interrupted by a frameshift mutation. A diploid cell complement (HG002) again has both complete open reading frames (ORFs) and other copies with the frameshift or deletions. In searches among non-human primate sequences to assess the evolutionary history of ORF3, a > 93% conserved copy of the full sequence of the ORF, as well as copies with in-frame deletions, was found in bonobo, but only fragments homologous to the ORF were seen in chimpanzee, orangutan, and gorilla rDNA examined thus far. ORF3 was expressed as a V5-tagged chimeric protein in human kidney epithelial HEK293 cells, and both ORF3-V5 and endogenous ORF3 were detected with a newly generated antibody. The protein is found in both cytoplasm and nucleus. However, upon treatment of cells with RNase A, the protein is excluded from the nucleus, suggesting that it is in complexes with RNA. Although any function is currently unknown, the ORF3 protein is upregulated, speculatively associated with changes in chromatin, in viral-transformed HEK293 cells and in human diploid fibroblast cells rendered senescent by treatment with etoposide, ionizing radiation, or an oxidant (H_2_O_2_).

**Supplementary Information:**

The online version contains supplementary material available at 10.1007/s00239-025-10269-1.

## Introduction

Since the discovery (Birnstiel et al. 1966) of tandemly repeated ribosomal DNA (rDNA) units in eukaryotes, organized on the 5 acrocentric chromosomes in humans (Birnstiel et al. 1971; Long and Dawid 1980), the hallmark ribosomal (r)RNA transcript is a *45S* pre-RNA that is processed to yield *18S*, *28S*, and *5.8S* rRNAs. In the intervening decades, several small noncoding RNA products from the intergenic spacer region have been reported (Agrawal and Ganley 2018), and a long antisense transcript, *PAPAS*, transcribed by RNA polymerase II, has been found to regulate the synthesis of *45S* rRNA via H4K20 trimethylation and transcriptional silencing (Bierhoff et al. 2014, 2010). However, it was widely believed that no protein-coding RNA was transcribed from the rDNA repeats. This notion was reinforced by the discovery that a copy of the *CDC27* gene in the “spacer” region of the rDNA units was a non-transcribed pseudogene. However, this view is being revised by the recent discovery of two transcripts with open reading frames (ORFs) transcribed from spacer rDNA, *ORF1* and *ORF2*, encoded in the *PAPAS* lncRNA (Feng et al. 2023) (see Discussion). It should also be noted that another transcript, TAR1, has been reported as encoded in 25S yeast rRNA, from which it is transcribed opposite to the direction of 25S rRNA (Coelho et al. 2002) and is proposed to suppress replication of petite mitochondria (Poole et al. 2012).

To investigate the variation among rDNA repeats, we recently sequenced 19 bacterial artificial chromosomes (BACs) containing rDNA repeat units from human chromosome 21, and 3 from human chromosome 22, which we had isolated by transformation-associated recombination (TAR) from mouse–human hybrid cell lines bearing a single copy of human chromosome 21 or 22 (Kim et al. 2018, 2021). Sequence analysis revealed a previously unreported potential ORF of 190 amino acids in the spacer region of multiple repeats, on the strand opposite the strand coding for pre-rRNA. We report analyses showing that the complete ORF is seen in human rDNA loci; and by comparison to sequences from non-human primates, a fully homologous sequence was only found in a bonobo sample, with conservation of 177/190 amino acids (93% homology at the predicted protein level); only fragmented portions of homology were found in available sequences from other non-human primates—chimpanzee, gorilla, and orangutan.

To begin to investigate whether this third ORF in rDNA is expressed in cells and has any function, we raised rabbit antibodies against the putative protein and found evidence that it is expressed in the nucleus of HEK293 cells but is released to the cytoplasm after RNase treatment. We further found that its expression levels did not change when RNA polymerase I was inhibited. Inhibition of RNA polymerase II unexpectedly did not alter the gene expression by much, but inhibition of RNA polymerase III with only known inhibitor CAS 577784-91-9 failed to show inhibition of control 5S RNA, so the results were inconclusive. Further, senescent human fibroblasts and stress-induced senescent cells showed higher ORF3 expression compared to replicating cells.

## Results

### Identification of Human-Specific Open Reading Frame in rDNA

**Fig. 1:**
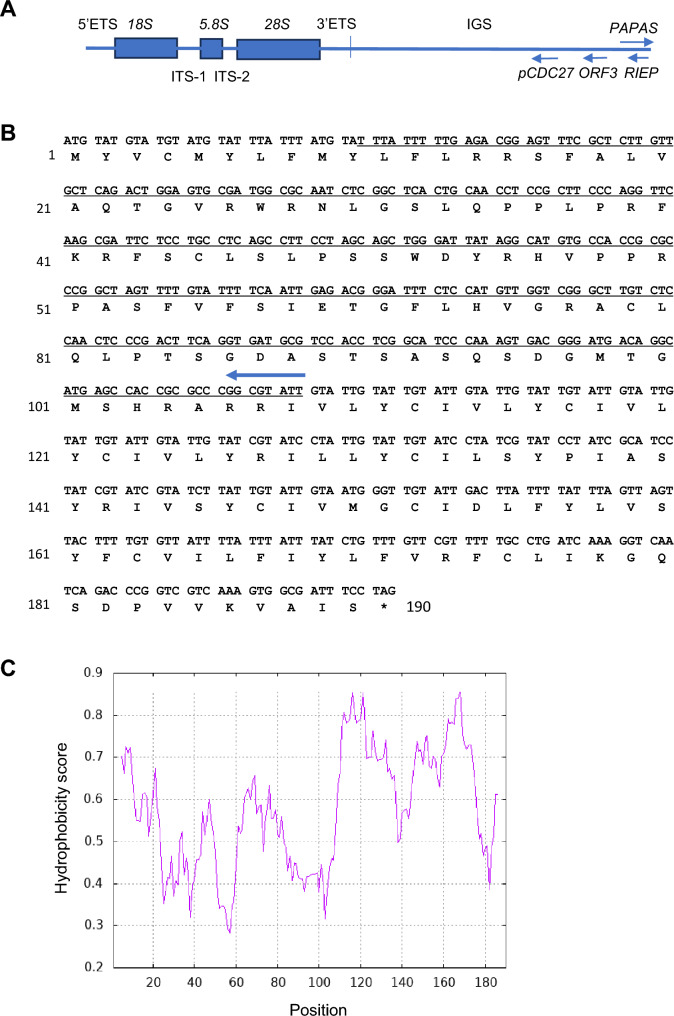
Schematic of ORF3 position in IGS, ORF sequence, and hydropathy plot. **A** Schematic representation of an rDNA unit with *18S*, *5.8S*, and *28S*, and 5’ and 3’ ETS sequence positioned along with the locations of lncRNA *PAPAS* (Bierhoff et al. 2014, 2010), *RIEP*, *ORF3,* and pseudogene *Cdc27*. **B** The translated DNA sequence for the open reading frame of 190 amino acids. An AluSx sequence insertion is underlined, and an arrow shows the direction of AluSx insertion. **C** Hydropathy plot based on the Kyte and Doolittle analysis (Kyte and Doolittle 1982), showing the hydrophobic nature of the protein on a 0–1 scale. The protein is highly hydrophobic in the N-terminal 10 amino acids and toward the C-terminal end starting at amino acid 110. The portion between those two segments precisely comprises the AluSx insertion, which consists of a relatively hydrophilic portion of the protein.

In the intergenic spacer regions (IGS) of sequenced BACs isolated from A9 mouse:human hybrid cells containing human chromosome 21, we found that 9 of 19 BACs contained rDNA units with ORFs capable of coding for a 190 amino acid protein (~ 22 kDa), using the EMBOSS Transeq program (https://www.ebi.ac.uk/jdispatcher/st/emboss_transeq). Like pseudogene *pCDC27* and ORF1, also called *RIEP* (Feng et al. 2023), ORF3 is encoded on the opposite strand from the pre-rRNA; their relative positions are indicated in Fig. 1A, and the full-length imputed protein sequence is shown in Fig. 1B. Among the other ten sequenced rDNA clones, two had a 5-base pair (bp) insertion in the ORF sequence and the other eight had a 5-bp deletion, all of which would be expected to lead to premature termination and thus shorter putative protein products with sizes of approximately 15.1 and 15 kD, respectively (S1 Figs. 1A and B, respectively, show nucleotide and corresponding protein alignments for all clones). The rDNA repeat-containing TAR clones from mouse:human hybrid A9 cells with human chromosome 22 contained only the version with the 5-bp insertion.

The sequence is notable in that it includes an AluSx (Smit et al. 1996–2010), a highly repetitive sequence inserted into the rest of the ORF (underlined in Fig. 1B). The encoded putative protein sequence is thus highly hydrophobic, with an overall hydropathic index of 0.708 on a scale of 0–1 (Kyte and Doolittle 1982). Furthermore, the protein is amphipathic: The segments outside of the Alu sequence (residues 1–10 and 110–190) are very hydrophobic, whereas the Alu sequences encode a hydrophilic region (residues 11–109; Fig. 1C) with 13 of the total 18 positively charged and 3 of the 6 negatively charged amino acids. The hydrophobic moiety includes an unusual repeated hydrophobic motif of 3 units of amino acids IVLYC and its variants IVLYR and ILLYC. We have named the coding sequence ORF3.

Alphafold program prediction (Jumper et al. 2021) of the secondary structure of the putative protein encoded by the complete ORF shows that it can potentially fold into 5 alpha helices spanning residues 2–30, 62–81, 104–134, and 144–180, followed by a very short helix between residues 183–187, with confidence scores (pLLD) between > 50 and < 70. The predicted possible alpha helical segments are linked by potential structurally disordered regions. An mp4 graphic version of the predicted possible structure is presented in Supporting Information Fig. S2.

### Characterization of *ORF3* mRNA by Northern Blot Analysis and 5’ RACE

**Fig. 2:**
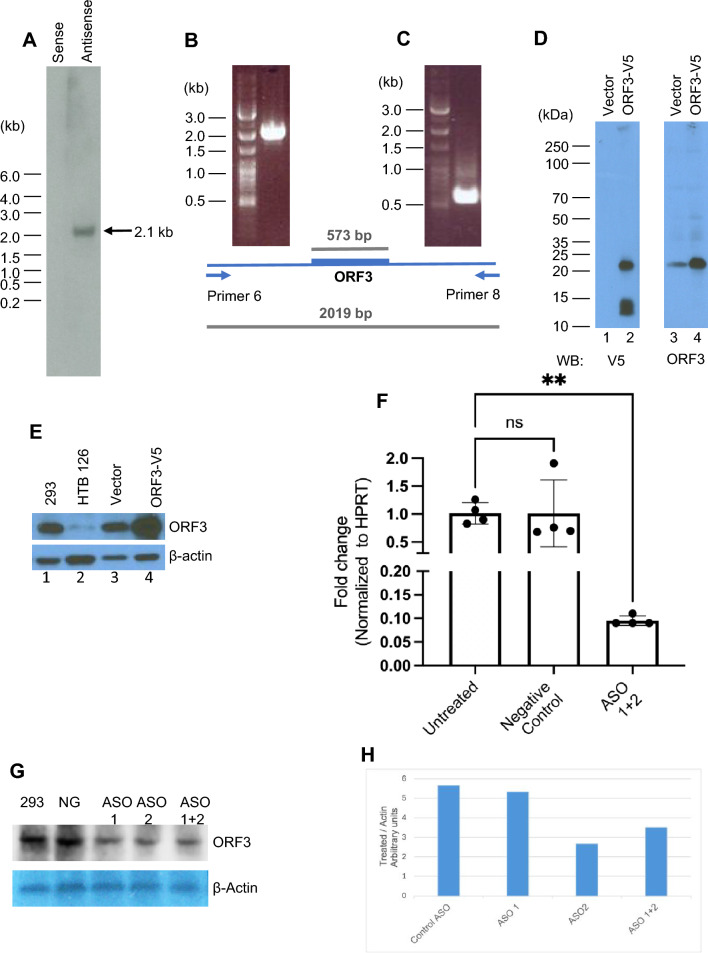
Transcription and translation analysis of *ORF3* mRNA. **A** Northern blot analysis of total RNA from HEK293 cells probed with sense and antisense oligonucleotides (see Supplementary Table 1 for oligo sequences). **B** Product of polymerase chain reaction with upstream primer 6 and downstream primer 8 from cDNA synthesized from total RNA HEK293 cells, resulting in a 2.1-kbp band on agarose gel. **C** PCR amplicon using primers spanning ATG start codon to TAG termination codon (Supplementary Table 1), yielding a diagnostic product band of 573 bp spanning the ORF and visualized on an agarose gel. Below the gel images is a schematic of the positions of primers 6 and 8 and ORF. **D** In HEK293 cells transfected with an empty vector (lanes1 and 3) or a vector expressing V5-tagged ORF3 (lanes 2 and 4), expressed proteins were detected by Western blot analysis using anti-V5 or anti-ORF3 antibodies. The V5-tagged ORF3 and the endogenous ORF3 are seen. **E** Western blot analysis of ORF3 in untransfected HEK293 cells, HTB126 cells, empty vector-transfected HEK293 cells, and ORF3-V5 transfected cells, using anti-ORF3 antibody in lanes 1, 2, 3, and 4, respectively. **F** *ORF3* mRNA levels in HEK293 cells 72 h after transfection of antisense LNA, as detected by RT-qPCR analysis. **G** ORF3 protein levels in HEK293 cells 72 h after transfection of antisense LNA, as detected by Western blot analysis. Lane 1 from the left, protein from untreated HEK293 cells; lane 2, cells transfected with control ASO (NG); lanes 3 and 4, with 2 independent ASO (1 and 2); lane 5, cells transfected with ASO 1 and 2 together. H) Plot of Image J analysis of expression levels of ORF3 normalized to Actin levels. Lanes are as in G.

Northern blot analysis was performed to assess whether the *ORF3* sequence is transcribed in vivo, starting with total RNA from human embryonic kidney HEK293 cells. The sense and antisense oligo probes used for hybridization were designed from the unique region in the ORF; sequences are listed in Supporting information Table 1. Figure 2A shows a ~ 2 kb sense strand band following hybridization with the antisense oligo, while the sense oligo yielded no detectable signal. Furthermore, when we isolated Poly(A)^+^ RNA and tested for the presence of transcript by Northern hybridization, no signal was seen, suggesting that this transcript lacks Poly(A) (data not shown).

To detect the 5’ and 3’ limits of the transcript, a set of primers upstream and downstream of the ORF, covering a span of 7 kb, were designed in unique sequence regions of the intergenic spacer (see Supplementary Table 1), inferred by repeat masking of the sequence using the Repeat Masker program (Smit et al. 1996–2010). Total RNA from HEK293 cells was used to synthesize cDNA using random primers (Materials and Methods). With this cDNA as template, upstream primers in combination with downstream primers were analyzed for the presence of a PCR product in agarose gels.

Figure 2B shows the presence of a 2,019 bp band produced when upstream primer 6 and downstream primer 8 were used for amplification. The resulting product was further tested by PCR for the presence of coding sequence, using a forward primer starting at ATG and a reverse primer starting at TAG (primer sequences are listed in Supporting information Table 1), yielding the expected 573 bp product in agarose gels (Fig. 2C). We found that the 5’RACE product obtained from downstream primer 8 in combination with upstream primer 6 also yielded the diagnostic 573-bp product. The 2019 bp sequence between upstream primer 6 and downstream primer 8 thus matches the transcript size determined by Northern hybridization. This product was sequenced to confirm the presence of the 190 amino acid ORF. Because the immediate region beyond these limits consists of repeated sequences that are therefore not amenable to unique primer design, it was not possible to test further for the exact 5’and 3’ ends of the transcript; however, because the products obtained from primers outside these limits failed to yield the diagnostic 573 bp product, we infer that the sequence extends further very little if at all.

### ORF3 is Constitutively Expressed in Cells

To further investigate the localization of ORF3, we tagged the nucleotide sequence with a V5 epitope sequence and cloned and expressed it in HEK293 cells from a CAG promoter. The expressed protein was detected by Western analysis (Fig. 2D) using an anti-V5 antibody. To detect the endogenous protein, we sought to express and purify the full-length protein in *E. coli* to raise antibodies, but these efforts failed because the full-length protein was toxic to the bacteria. However, a GST-tagged version lacking the N-terminal 21 amino acids was successfully expressed. The truncated protein was used to raise antibodies in rabbits that were tested against lysates of HEK293 cells transfected with the ORF3-V5 clone (Fig. 2D). The expected full-size 20 kDa band was detected both by anti-V5 and anti-ORF3 antibodies, further supporting the inference that the protein is expressed in these cells. [A smaller ~ 12 kDa protein band was only detected by anti-V5 antibody, suggesting that it is a cleaved fragment lacking the epitope(s) recognized by anti-ORF3.]

We confirmed that untransfected HEK293 cells (Fig. 2E, lane 1) expressed the protein endogenously, as did an established cancer cell line, HTB126 (Fig. 2E, lane 2); HEK293 cells transfected with either empty vector or ORF3-V5 were included as controls. Together, these results support the inference that the *ORF3* embedded in the spacer region of rDNA is both transcribed and translated.

### ORF3 is a Relatively Stable Protein

To begin to analyze the stability and dispensability of ORF3, two independent antisense locked nucleic acid (LNA) oligos (ASO) (Supplementary Table 1) were designed in the unique region of the ORF coding sequence. Transfections with antisense ASOs effectively decreased protein levels in cells grown for 72 h (Fig. 2F) compared to control. However, levels were only moderately reduced compared to controls even after 72 h incubation with ASOs (Fig. 2G), suggesting that the protein is relatively stable. At the levels of inhibition seen, no visible changes were observed in the appearance of cells or their growth rate (data not shown). Whether longer silencing interventions or silencing by other cell treatments might result in an observable phenotype remains to be determined. Figure 2H shows the levels of protein analyzed by Image J, normalized to actin levels and suggests that inhibition by ASO 2 was more effective compared to ASO 1.

### Effect of RNA Polymerase Inhibitors on ORF Expression

**Fig. 3:**
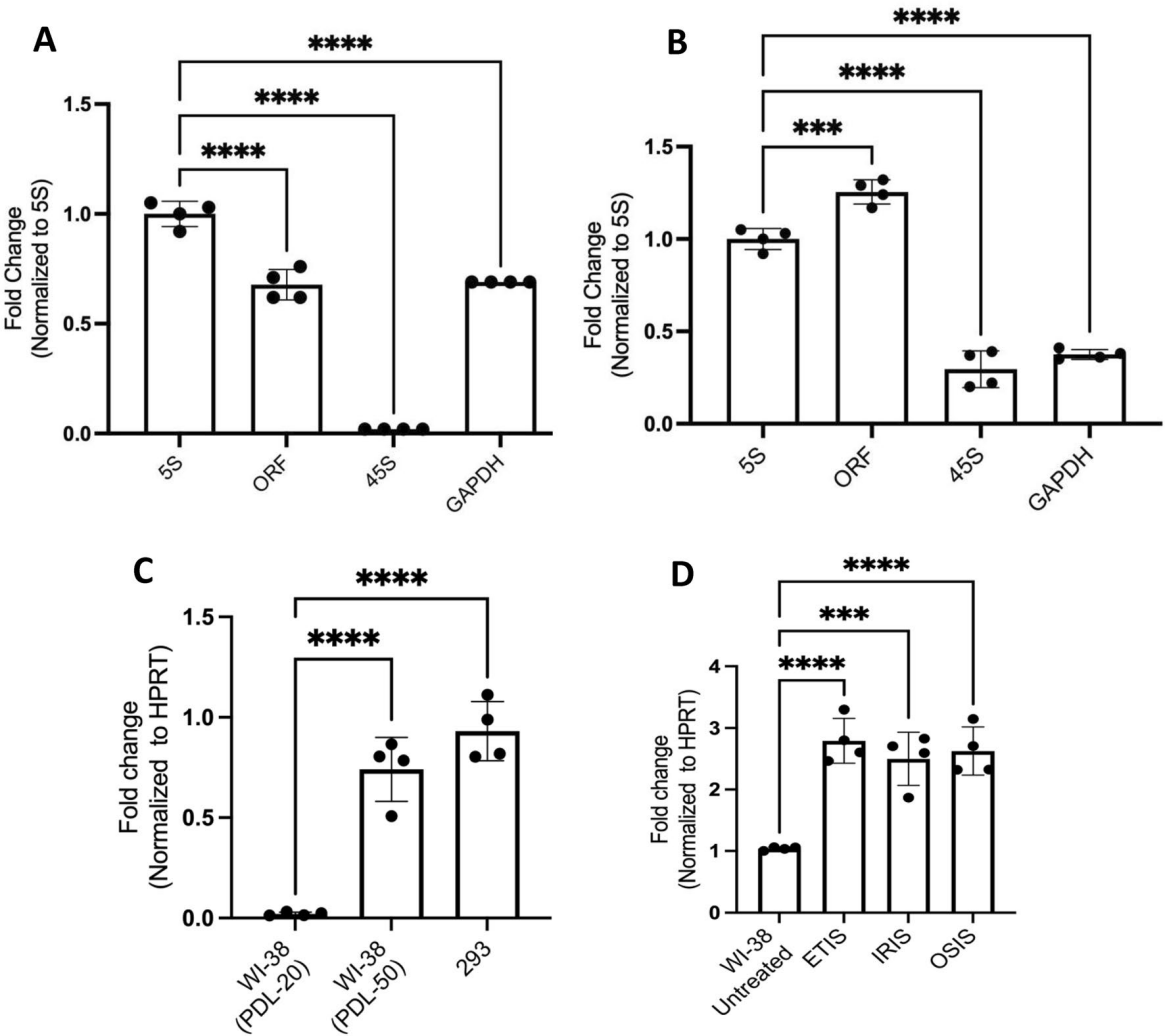
Effect of POL I and POL II inhibition on *ORF3* transcription and mRNA levels in replicative and senescent cells. **A** RT-qPCR analysis of the levels of *45S* rRNA, control *5S* RNA, and *ORF3* RNA 24 h after treatment with the RNA Polymerase I inhibitor BMH1. **B** RT-qPCR analysis of the levels of *45S* rRNA and *ORF3* RNA 24 h after treatment with the RNA Polymerase II inhibitor DRB. **C** RT-qPCR analysis of *ORF3* mRNA levels in replicating WI-38 cells (passage 20) senescent WI-38 cells (passage 50) and HEK293 cells. **D** *ORF3* mRNA levels in WI-38 cells that were proliferating or rendered senescent by treatment with etoposide (etoposide-induced senescence, ETIS), ionizing radiation (IRIS), or H_2_O_2_-triggered oxidative stress (OSIS)

Total RNA was isolated from HEK293 cells that were treated with either RNA Polymerase I inhibitor BMH21 (Jacobs et al. 2022), RNA Polymerase II inhibitor DRB (Baumli et al. 2010), or RNA Polymerase III inhibitor CAS 577784-91-9 for 24 h (Sigma-Aldrich) (Wu et al. 2003). After preparing cDNA using random primers, we quantified the level of inhibition of *ORF3* mRNA by RT-qPCR analysis. Diagnostic primers were designed from the C-terminal unique sequence region of the protein (Supporting Information Table 1). The results show that BMH21 efficiently repressed the production of *45S* RNA compared to untreated control (Fig. 3A), but it did not affect the levels of *ORF3* mRNA, suggesting that RNA Polymerase I does not transcriptionally induce *ORF3* mRNA. Pol II inhibitor DRB also showed no inhibition; rather, the expression of ORF3 was increased in the presence of the inhibitor (Fig. 3B, see “Discussion”). Because Pol II inhibition also leads to inhibition of GAPDH formation, Pol II (and PolI) inhibitor results were normalized to 5S RNA levels. For reference, we have included GAPDH-normalized plots as Supporting Material Figures S4A and B. This also suggests that there is an increase in the ORF transcription. Although CAS 577784-91-9 had been reported as an inhibitor for Pol III (Wu et al. 2003) it did not show the expected strong inhibition of 5S RNA formation and also failed to inhibit transcription of ORF3 effectively in HEK293 cells, so that transcription by Pol III remains a possibility (data not shown; see Discussion).

### ORF3 Transcription Increases in Senescent Fibroblasts and Transformed Cells

To study the expression of *ORF3* in normal cells, we extracted RNA from replicating human diploid WI-38 fibroblasts (passage 20) and senescent WI-38 fibroblasts (passage 50). RT-qPCR analysis revealed that relative to proliferating WI-38 fibroblasts, *ORF3* mRNA was much higher in senescent WI-38 cells > 95%, and for comparison we showed its expression in HEK293 cells (Fig. 3C). In WI-38 fibroblasts rendered senescent by treatment with etoposide, ionizing radiation (IR), or an oxidant (H_2_O_2_) (Anerillas et al. 2022), *ORF3* mRNA levels increased 2.5-fold, as measured by RT-qPCR analysis (Fig. 3D). These results indicate that senescence and transformation of cells, two processes in which cells have corresponding translation adaptations, induce the expression of the *ORF3* transcript.

### In Vivo Localization of ORF3

**Fig. 4:**
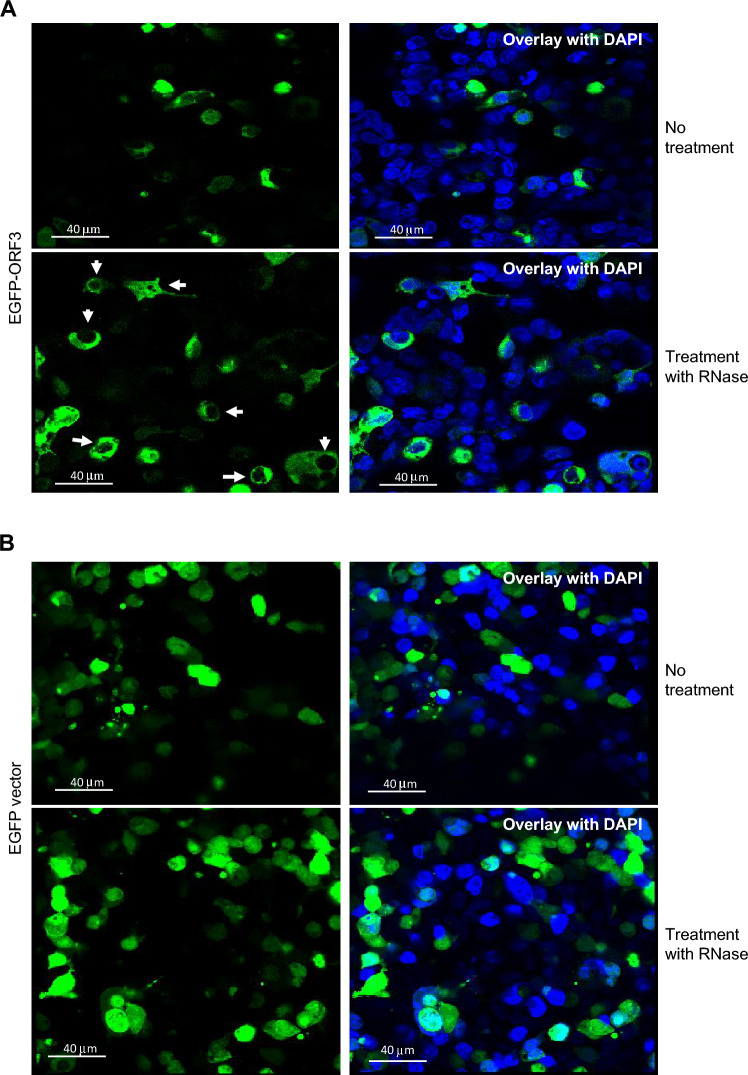
Immunofluorescent detection of chimeric EGFP-ORF3 in HEK293 cells. HEK293 cells transfected to express EGFP-ORF3 (**A**) or EGFP vector alone (**B**) and fluorescent signals were detected 72 h later. As indicated, cells were either left untreated or treated with RNaseA before fixation. Where indicated, DAPI (Blue) was used to visualize nuclei.

To assess in vivo localization of the protein, the ORF3 was fused to EGFP and the chimeric EGFP-ORF3 protein was expressed in HEK293 cells. EGFP fluorescence was assessed 72 h later and was found to be both nuclear and cytoplasmic (Fig. 4A); however, upon treatment with RNase, the fluorescent protein was released from the nucleus and migrated to the cytoplasm (‘Treatment with RNase’). Control transfections with a vector containing EGFP alone did not show this change (Fig. 4B). This suggested that the localization of the protein in the nucleus is dependent upon the presence of RNA or an interaction with a protein associated with RNA. Consistent with these results, fractionation of lysates (Senichkin et al. 2021) of HEK293 cells expressing ORF3 showed roughly comparable amounts of the 20 kDa band in Western blots (as in Fig. 2D) in both nucleus and cytoplasmic fractions, as shown in Supporting Material Figure S4C.

### Distribution of ORF3 in the CHM13 Full Chromosome Complement

A search in the GenBank database for ORF3 identified one match, annotated as derived from an unplaced contig (CKC 03773, Bioproject PRJEB2480) of *Streptococcus pneumoniae* DNA isolated from a human nasopharynx sample. The sequence does not, however, appear in the complete sequence of that bacterium, and a comparison of the reported DNA sequence for CKC03773 to human rDNA (KY962518) revealed that this unplaced contig is instead homologous to the human rDNA IGS sequence. It thus appears to have been misannotated in the bacterial sequence database, likely resulting from contamination by human DNA in the isolate from human nasopharynx [GenBank has recently added a note that this sequence does not belong to *S. pneumoniae*.].

**Fig. 5:**
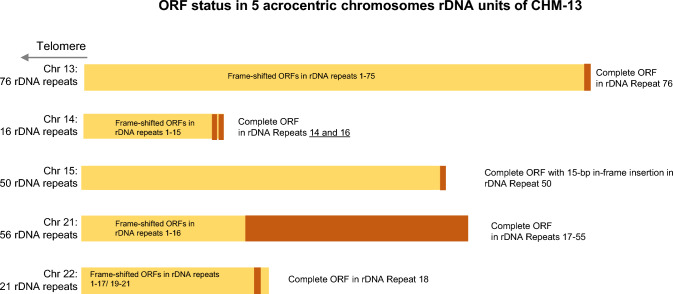
ORF3 ORF distribution in CHM13 acrocentric chromosomes. Each rDNA unit is screened for the presence of ORF; brown bars indicate repeat number(s) containing an ORF. Gold bars indicate the locations of ORFs with deletions leading to frame shift and truncation of the open reading frame. Chromosome 21 contains 39 ORFs, the bulk of the total. As indicated, chromosomes 13, 15, and 22 each contain one ORF, with two ORFs on chromosome 14 in the most centromeric rDNA units.

With the release of telomere-to-telomere sequence data for a complete haploid chromosome complement from CHM13 (Nurk et al. 2022), ribosomal DNA sequences on the acrocentric chromosomes are resolved. We could thus examine the genomic rDNA repeats for ORF3 sequence in that complete genome. Complete ORF3 copies are seen in 19% of the total of 219 rDNA repeats represented in the CHM13 genome; 39 of 43 of them are on chromosome 21 in consecutive rDNA repeats 17–55 of the 56 complete rDNA units, numbered from telomere to centromere on the acrocentric short arm. [We note, however, that this region of the CHM13v2.0 assembly is uncertain, and although the copy number of these units should be accurate, their consecutive ordering may require some revision on further sequencing (Nurk et al. 2022)]. By contrast, chromosomes 13, 15, and 22, each contain only 1 complete ORF unit among a total of 76, 50, and 21 rDNA units, respectively (Fig. 5). Chromosome 14 has 2 copies of ORF3 in rDNA units, 14 and 16 of its 16 rDNA units, with repeat 15 and the rest of the units having a 5-base pair insertion, while on chromosome 22, a copy of ORF3 is in the 18th of the 21 rDNA repeats. In further analyses, the one ORF3 repeat on chromosome 15, in its 50th rDNA unit, has an extra 15 base in-frame insertion coding for an extra unit of the IVLYC repeat unit. In addition, it has a mutation L > S at amino acid 11 due to a T > C transition at base 32 and an L > V change at amino acid 134. The L > V change is found on all complete repeats except for the ORF3 copy on chromosome 22, which has the same leucine amino acid seen in the TAR cloning isolates. The individual ORFs on chromosomes other than 21 are all toward the centromeric end of their complement of rDNA repeats (see below).

Compared to the ORFs from TAR clones from mouse hybrid cell line A9 containing human chromosome 21, the ORFs in CHM13 also all had a variant C > G residue, leading to an amino acid change L > V at position 129. A survey of 48 samples from HPRC consortium (Liao et al. 2023) from a range of populations (including African, East and South Asian, American, and Caribbean) from HG002 (Rhie et al. 2023) found that the major version of the protein contained the L > V at position 129 ranked at 25%, with a range of 30–70 copies per haploid genome. The amino acid sequence matching the TAR clone version was found in 13%. We also found other versions of the protein with 2 or more base changes at different positions. By contrast, the clones from the chromosome 21 in the mouse A9 hybrid cells, derived from fibroblasts from a Japanese individual, lacked this change, showing that other variants are distributed in the human population. Also, of note is that the samples from the HPRC consortium showed frame-preserving changes at 43% and ORFs with 2 or more changes occurring in 19% (see Methods). A list of ORF nucleotide sequences in Fasta format with 1, 2, or more than 2 amino acid changes is provided in Supporting Information files (1change.txt, 2change_ungapped.txt, and 2pluschange_ungapped.txt that can be visualized in Jalview) (Waterhouse et al. 2009).

In addition, the ORF3 unit on chromosome 15 has a 15 bp insert that leads to an in-frame 5-amino-acid insertion in the sequence. The remaining rDNA units in all chromosomes have either 5 bp deletions or 10 bp insertions that lead to premature stop codons, as illustrated in Supplementary Figs. 1A and B. All those rDNA repeats contain the same nearby upstream and N-terminal sequence of ORF3, but we have not explored whether non-full-length versions of the ORF are expressed. In the other completely sequenced mammalian genomes, mouse (Liu et al. 2024), and our own TAR-cloned mouse isolates (Kim et al. 2024), no sequence corresponding to ORF3 was detected, and the final organization of the expressed gene seems to have occurred only in higher primates.

### Extent of Conservation of ORF3 in Non-human Primates

The ORF sequence from the analysis above was used to search rDNA HiFi data of non-human primate sequences (Makova et al. 2024) from *Gorilla gorilla* (gorilla, KX61887.1), *Pan troglodytes* (chimpanzee, KX061886.1), *Pongo abelii* (Sumatran orangutan, KX061888.1), and *Pan paniscus* (bonobo, GCA_029289425.2) (in GenBank and in our own unpublished sequence from TAR rDNA clones from gorilla, chimpanzee, and orangutan derived from cell lines from Coriell Institute for Medical Research AG20600, GM03448A, AF20604). Although nucleotide sequences matches were found in these species, the ORF portion was fragmented compared to human rDNA sequence, with a combination of deletions, insertions, and single base changes in all non-human primates tested except for bonobo. Supporting Information Fig. S3A shows the alignments between the ORF3 transcript and reported sequences from rDNA of the 4 non-human primates. Gorilla, chimpanzee, and orangutan sequences lack a start codon corresponding to the start site in human, and there is thus no corresponding expected protein product. but a start codon is present in bonobo, and a representative nucleotide homology and translation comparisons are shown in Supporting Information Fig. S3A and S3B.

The ORF3 sequences in bonobo are found on its chromosomes 14, 15, 17, 22, and 23 (HSA13, HSA14, HSA18, HSA21, HSA22). A survey of currently available genomic bonobo sequence (GCA_029289425.2) assembly on these chromosomes using the translated representative sequence shown in Supporting Information Fig. S3B was done to assess the copy numbers of the ORFs. The analysis showed that there are about 40 copies of ORF3 coding sequence per haploid genome, based on the analysis of raw reads of an estimated 72 copies of ribosomal units per diploid genome. The conservation between ORF3 from the A9 hybrid cell line and that of bonobo is 93% at the protein level (170 of 190 amino acids) (see Methods). In comparison with the limited number of rDNA repeats in the assembled sequence in the database (GCA_029289425.2), as in CHM13, several frame-shifted copies were found on all the chromosomes containing rDNA units. In addition, except for the ORF on chromosome 17, the sequence deviated from the representative copy used for comparison, with all other copies carrying additional mutations. The two copies on chromosome 14 had C > R at position 122, P > R at position 131, and L > V at position 133. The three copies on chromosome 15, 22, and 23 had shortened versions of the putative protein due to absence of one or two in-frame hydrophobic ‘IVLYC’ repeat units. Also, two copies analyzed from chromosome 23 had amino acid variants L > V at position 134 and M > I at position 167 in one hand and C > R at 127 and L > V at position 135 on the other hand.

In further analyses of chimpanzee and orangutan sequences, the distal “coding region” is interrupted by a 43-base deletion, and in gorilla, a 13-base insertion leads to a break in the comparison with ORF3. These interruptions are illustrated in Supplementary Fig. 3B, where the homology between ORF3 protein and the homologous sequence in non-human primates is illustrated. In gorilla, good homology starts at amino acid (a.a.) 13 and ends at a.a. 105 with a frame shift; the homology then picks up again from a.a. 139 until the end of the ORF sequence at a.a. 190, resulting in two putative “fragments.” In chimpanzee, the homology that begins at a.a. 14 is interrupted at the IVLYC hydrophobic region, where there is an expansion of this repeat in human, and continues to the end of the ORF at a.a. 190. In orangutan, a good homology starts at a.a. 67, with a frame shift in the hydrophobic region caused by a stop (*) codon next to “CCVV*” (see Fig. S3). The sequences available thus far suggest features of the evolution of ORF3 (see Discussion).

## Discussion

We report here that a coding sequence located in the IGS region in rDNA locus is transcribed and translated to yield a protein that we have called ORF3. The protein is characterized by the presence of 289 bp of AluSx sequence inserted between coding sequence nucleotide 31 and 319, flanked by unique sequences at the N- and C-termini. It is encoded by a~2,019 base long mRNA, as supported by Northern blot analysis and RT-qPCR assay, with verification that a PCR product derived from primers upstream and downstream of ORF contains the expected open reading frame and flanking sequence. The transcript is translated into a protein of 190 amino acids, as supported by an antibody raised against the protein.

Recently, a report described two other open reading frames, a short “ORF1” and a larger “ORF2” (Feng et al. 2023), both located within the lncRNA PAPAS sequence transcribed from the strand opposite transcription of 45S pre-rRNA (Bierhoff et al. 2014). The larger of the two, ORF2, encoding a 229-amino acid protein, RIEP, was shown to be induced by heat shock, and its product was shown to associate with the rDNA locus and to upregulate the levels of Senataxin, an RNA: DNA helicase that reduces the level of DNA damage during heat shock. In addition, RIEP was also shown to interact with two mitochondrial proteins, C1QBP and CHCHD2. A comparison of this ORF to the rDNA units from CHM13 (Nurk et al. 2022) shows the presence of a complete ORF in all rDNA units of chromosomes 14, 15, 21, and 22. However, on chromosome 13, only the last 3 rDNA repeats (repeats 74, 75, and 76, numbered from the telomere) contain a complete ORF; the other 73 rDNA units have a deletion of 19 bp, leading to termination of homology at amino acid 175 of RIEP. This 19-bp deletion was also included in rDNA reference sequence U13369 and revised rDNA reference KY962518. In this respect, RIEP is similar to ORF3, with several versions of the coding sequence observed: a complete ORF in some rDNA repeats and a form interrupted by deletions in other rDNA repeats. However, RIEP, unlike ORF3, has a complete homologous sequence in various non-human primates (Feng et al. 2023). [Further sequence comparisons to GenBank entries show complete homology of REIP to an annotated protein, CKC08924; but as seen for ORF3, this protein was again misannotated in the database, erroneously ascribed to *Staphylococcus pneumoniae*].

Based on currently available sequence, it is notable that there is considerable conservation of sequence, both upstream and downstream of the ORF in human and non-human primates, including the presence of an Alu sequence within a region homologous to ORF3. This suggests that the insertion of Alu in the rDNA spacer region had occurred, and sequences upstream and downstream of the ORF were also in place before the evolutionary separation of primates into human and non-human lineages. But we have thus far found that only human and bonobo have the start codon for residue 1 of ORF3, presumably arising from a rare mutation. The resultant uninterrupted sequence, and especially its repeated motif of 5 hydrophobic amino acids, is unique to date: conserved domain searches (Wang et al. 2023) found no other protein with such sequence.

The complete reading frame is present in all human samples thus far analyzed, though in a minority of human rDNA or bonobo repeats. But only a few rDNA units have been analyzed from other organisms, so that it is not presently excluded that other primates or even lower organisms may still prove to contain a complete ORF3 orthologue in some repeats. However, it is telling that all the rDNAs examined thus far in chimpanzee, gorilla, and orangutan lack a start codon in their partial ORF3 sequences and have other deficiencies, greatly strengthening the likelihood that the gene is conserved evolutionarily in bonobo and *Homo sapiens*, originating in a common ancestor.

An auxiliary question is how and when the postulated incorporation of the AluSx sequence into the amino acid sequence occurred during evolution. Since the pioneering discussions of Ohno et al. (1968), evolutionary biologists have accepted the notion that duplication and divergence yield genes with expanded and alternate functions. Another source of “duplicate gene source material” comes from exonization of Alu repeat sequences inserted into introns (Prey 2008; Sela et al. 2007; Zhang and Chasin 2006). *ORF3* supplies an example that probably occurred only once during evolution: an Alu sequence “jumped” into the possible ORF region in an rDNA spacer. From the current data, we can infer a possible course of subsequent events. The initial Alu insertion was out of frame with the adjacent coding sequence and lacked a start codon, as seen in non-human primates such as gorilla, chimpanzee, and orangutan. However, it then acquired and established coding capacity by additional mutations during evolution, as seen in bonobo and human, including the creation of the ATG initiating methionine codon. This version then putatively spread across human acrocentric chromosomes by intra-chromosomal gene conversion and inter-chromosomal exchange. Thus, on chromosome 21 of CHM13, functional ORFs were aligned in 39 consecutive rDNA units. But in other lineages, the distribution is likely to be quite different. One basis for variable distribution across acrocentric chromosomes can be inferred from evidence for exchange of ribosomal DNA as a block due to the presence of pseudo-homologous regions (PHR) between rDNA-containing chromosomes; exchanges between these regions would facilitate transfer of ribosomal units between chromosomes (Guarracino et al. 2023); and inversion of PHR in chromosome 14, with recombination between 13 and 14, can lead to Robertsonian translocations between them. The dynamics of *ORF3* copy number and distribution should become clear now that complete rDNA loci can be assembled with nanopore-based long-read sequences from families and different populations. Similarly, the evolutionary course and the frequency of specific variants should become clearer as more human and non-human rDNA repeats are assembled and analyzed. ORF3 variants may themselves provide useful lineage markers in future studies of human populations.

The transcription of ORF3 showed unusual features when we attempted to identify the RNA polymerase involved. It showed no change when RNA Polymerase I was inhibited. When Pol II was inhibited, the unexpected increase in ORF3 level may reflect greater chromatin access, possibly similar to the observation that when Pol II is inhibited, the loss of an r-loop shield formed in association with senataxin leads to Pol I producing sense intergenic sense RNAs (sincRNA) (Abraham et al. 2020). Pol III, the other candidate polymerase, is known to transcribe noncoding RNAs, but has not been reported to transcribe a gene encoding a protein, and in our case, Pol III inhibition by the only reported Pol III inhibitor, CAS 57784-91-9 (Wu et al. 2003), was ineffective in inhibiting transcription of either the control 5S RNA or the ORF. Thus, further study is needed to identify the polymerase involved in RBSM expression.

Given that only fragmented versions of ORF3 were identified in several non-human primates but a complete gene sequence is present in bonobo, any functional role of ORF3 would be limited to bonobo and human but remains conjectural. Suggestively, some of the cellular ORF3 was found in the nucleus, but the NoD program (Scott et al. 2011) found no nuclear localization signal protein in the gene. Thus, the protein apparently enters the nucleus bound to another factor, and because its nuclear localization was lost after RNase treatment, it is putatively bound to either RNA or an RNA-containing complex, a possibility that requires further investigation.

A further hint to a possible function is the low level of ORF3 in growing fibroblasts contrasted with its sharp increase in senescent cells; in this regard, it becomes a previously unrecognized part of the modified gene expression profile in senescent cells. However, its high level in a transformed cell line makes it more unusual. It may be relevant that in all these cases, substantial changes in chromatin structure and function occur associated with modified growth state; further studies of its subcellular localization and partners in likely RNA:protein complexes should reveal more about its possible function. But at this time, the possibility remains that *ORF3* represents an ongoing experiment of evolution, involving the formation of a novel protein without an as-yet evolved function.

## Materials and Methods

### Cell Growth and Transfection

HEK293 (CCL-1573) and HTB126 cells were obtained from ATCC (Manassas, VA, USA) and cultured in the DMEM medium with 10% fetal bovine serum (FBS; Atlanta Biologicals) in 5% CO_2_ at 37 °C, as advised by ATCC. All media were purchased from Invitrogen, and cDNA plasmid constructs were transfected into HEK293 cells with Lipofectamine 2000 Plus reagent (Invitrogen) following the manufacturer’s protocol. Following culture for 72 h, cells were lysed in lysis buffer (Tris 50 mM, pH 7.4, NaCl 150 mM, MgCl_2_, 2 mM, EDTA 2 mM, 0.1%Triton X-100 and 0.1% Igepal CA-630; ThermoFisher). After centrifugation at 13,000 rpm for 10 min at 4 °C, protein expression was determined by Western blot analysis (below). WI-38 fibroblasts were either proliferated or rendered senescent by replication, treatments with etoposide, irradiation, or oxidative stress with hydrogen peroxide.

### Northern Blot Analysis

Total RNA was isolated from proliferating or senescent WI-38 cells, or HEK293 cells, using TRIzol reagent (15596026, ThermoFisher). For Northern blot analysis, 5 µg of total RNA from HEK293 cells was fractionated in a 1.2% agarose gel in MOPS buffer with 2.2 M formaldehyde (He and Green 2013), along with RNA markers (Thermofisher, AM7150), and transferred to nylon membranes by capillary transfer overnight in 10 × SSC buffer. The bound RNA was crosslinked in a Stratalinker by UV irradiation with 1800 Joules/cm^2^. Two lanes containing RNA were separated by slicing the membrane. The two strips of membrane were independently prehybridized with 0.2 mg/ml denatured salmon sperm DNA for 2 h in 10% dextran sulfate, 1 M NaCl, and 1% SDS. Twenty-five pmoles each of sense and antisense oligos (Sequence in Supplementary Table 1) were end-labeled using 50 pmol of γ-^32^P[ATP] (Perkin Elmer Health, NEG002A250UC) and 10^8^ counts per minute (CPM) of each oligonucleotide was added separately to each strip in the hybridization mix and incubated overnight at 55 °C. The membranes were removed and washed briefly with 6 × SSC and 1% SDS at room temperature and with two successive washes at 50 °C with 4 × SSC and 2 × SSC with 1% SDS for 30 min each. A final wash with 0.2 × SSC with 1% SDS was performed at room temperature for 10 min, and the dry membrane was exposed to Kodak XO-MAT AR film overnight at − 80 °C and developed.

### Tagged Protein Construction

The ORF3 coding sequence was amplified using PCR analysis from BAC JH14 (MF164261.1); forward and reverse primers used for amplification are listed in Supplementary Table 1. The PCR product was cloned into the pcDNA3.1-V5-His vector (Thermofisher). Isolated clones were sequenced to verify the integrity of the sequence. To construct a pEGFP plasmid, ORF3-V5-His plasmid was digested by Hind III and EcoR5. The Hind III-EcoR5 fragment containing the ORF3 coding region was cloned into the pEGFP-C2 vector (Clonetech) digested by Hind III and SmaI. 

### Anti-ORF3 Antibody Production

A GST-fusion protein containing amino acids 22–190 of ORF3 was expressed in bacteria, purified, and used as an antigen to produce anti-ORF3 antibody in rabbits (Proteintech).

### Subcellular Fractionation

Cell fractionation was done as previously explained (Senichkin et al. 2021). Briefly, all procedures were carried out on ice and centrifugation was performed at 4°C. After the culture medium was aspirated from the plate, cells were washed once with ice-cold phosphate buffer, collected by scraping, and resuspended in hypotonic buffer (5 × cell volume of 20 mM Tris–HCl, 10 mM KCl, 2 mM MgCl_2_, 1 mM EGTA, 0.5 mM DTT, 0.5 mM PMSF) containing 0.1% NP40 and incubated for 3 min. The cells were homogenized using a Potter-Elvejehm homogenizer with 15 strokes of the pestle, and the solution was centrifuged to pellet nuclei (500 × g, 5 min). The supernatant was clarified at 15,000 RPM for 3 min to pellet debris. The nuclear fraction was resuspended in isotonic buffer (20 mM Tris–HCl, 150 mM KCl, 2 mM MgCl_2_, 1 mM EGTA, 0.5 mM DTT, 0.5 mM PMSF) with 0.3% NP40 for 5 min and then centrifuged (500 × g, for 3 min). The supernatant was collected for further analysis. The cytoplasmic and nuclear fractions were mixed with SDS gel loading buffer, boiled for 5 min, and electrophoresed on a 4-to-15% gradient gel before loading them on SDS–polyacrylamide gel and size-fractionating them at 80 V for 2 h.

### Immunoprecipitation and Western Blot Analysis

HEK293 cells were transfected with *ORF3*-*V5*, and 72 h later, cells were solubilized in IP buffer (Tris 50 mM, pH 7.4, NaCl 150 mM, MgCl_2_, 2 mM, EDTA 2 mM, 0.1%Triton X-100 and 0.1% Igepal CA-630) on ice for 15 min and then passed through a 26-gauge needle three times to disperse aggregates. The clarified lysates were centrifuged at 13,000 RPM at 4 °C for 15 min, and supernatants were incubated overnight with goat anti-V5 agarose beads (S190-119, Bethyl lab) at 4 °C. The beads were washed 5 times, 15 min each, with IP buffer at 4 °C with rotation. The final immunoprecipitation reactions were separated by electrophoresis through 4% to 15% Tris–glycine and SDS-containing polyacrylamide gels (SDS-PAGE). The protein was then transferred to nitrocellulose membrane for Western blot analysis.

The membrane was blocked with 5% nonfat milk at room temperature for 1 h and incubated with the appropriate primary antibody at 4 °C overnight. Blots were washed 5 times with TBS buffer prior to incubation with HRP-conjugated goat anti-rabbit or anti-mouse IgG (GE healthcare) as required for 45 min, followed by washing again in TBS buffer. Immunoreactive bands were detected with the enhanced chemiluminescence reagent (Amersham) and imaged by exposure to GenHunter Blue film.

Primary antibodies used in this study include β-Actin [Santa Cruz (SC-8432)], V5 [ThermoFisher (46-0705)], and Anti-ORF3.

### Reverse Transcription (RT) Followed by Quantitative (q)PCR Analysis

From total RNA isolated as above, first-strand DNA was reverse-transcribed (RT) from 2 µg RNA with random primers using a SuperScript IV kit (18091050, ThermoFisher). The resulting first-strand DNA was then used for quantitative (q)PCR analysis on a QuantStudio 5 (Applied Biosystems) instrument or CFX Connect Real-Time System (Bio-Rad) using IQ SYBR® Green Supermix (Bio-Rad) with *ORF3*-specific primers; amplification of human *HPRT* mRNA or *GAPDH* mRNA was included in control reactions. The primer sequences are listed in Supplementary Table 1.

HEK 293 cells were treated for 24 h before RNA isolation with either 1 μM Pol I inhibitor BMH21 (Jacobs et al. 2022), 50 μM Pol II inhibitor 5,6-dichloro-1-β-D-ribofuranosylbenzimidazole (Baumli et al. 2010) (DRB), or 60 μM Pol III inhibitor CAS 577784-91-9 (Wu et al. 2003). First-strand DNA from RT reaction was diluted 1:20 and used in qPCRs. The amplification conditions were as follows: 95 °C for 4 min, followed by 45 amplification cycles at 95 °C for 15 s, 60 °C for 25 s, and 72 °C for 30 s. The levels of *GAPDH* mRNA and *45S* rRNA were included as controls for sample amount. Expression levels were calculated using the 2^−ΔΔCT^ method and normalized to control expression levels of *HPRT* mRNA for experiments with ASO, etoposide-treated, and proliferating and senescent WI-38 cells; *GAPDH* mRNA was used in experiments involving inhibitors of RNA polymerases I, II, and III.

### Imaging of Chimeric EGFP-ORF3 in Live Cells

HEK 293 cells were transfected with ORF3-GFP or control GFP plasmid; 72 h later, cells were treated with RNase A (EN0531, ThermoFisher) at 0.1 mg/ml for 10 min at room temperature. Treated and untreated cells were examined, and images were captured in a Keyence Fluorescence Microscope at 40× magnification.

### ORF3 Analysis in Human and Non-human Primate Sequencing Data

We analyzed HiFi sequencing data from 48 samples from the HPRC consortium (Liao et al. 2023) (HG00423, HG00544, HG00609, HG00621, HG00642, HG00735, HG00738, HG00741, HG01099, HG01106, HG01175, HG01255, HG01258, HG01346, HG01496, HG01884, HG01891, HG01943, HG01952, HG01981, HG01993, HG02004, HG02132, HG02148, HG02280, HG02293, HG02300, HG02486, HG02559, HG02572, HG02602, HG02615, HG02622, HG02630, HG02647, HG02683, HG02698, HG02738, HG02886, HG03453, HG03540, HG03669, HG03710, HG03831, HG03927, HG04115, HG04184, HG04199) plus HG002 (Rhie et al. 2023). We also analyzed non-human primate HiFi data from Makova et al. (2024). We used the TAR-cloned amino acid sequence of ORF3 and ran BLAST v2.0.15 (Altschul et al. 1990) on the read sets with the command:

*blasx -db gene.blast -query < (bunzip2 -c $input.fastq.gz |seqtk seq -A -) -outfmt 6*

Any reads with a match over 85% identity and over 150 AA in length were re-aligned in nucleotide space with MUMmer v4 (Marcais et al. 2018) with the commands:

*nucmer –maxmatch –nosimplify gene.fna match_reads.fasta*

*delta-filter -q out.delta.*

*show-coords -lrcTH out.delta > out.coords*

We extracted the matching sequence in each read with a match over 85% identity and 550 bp and checked for an ORF using ORF Finder v0.4.3 (Wheeler et al. 2003) and the command:

*ORFfinder -ml 180 -n false -in < input sequences > -s 0 -out orf -outfmt 0*

The count was normalized by the haploid genome coverage (total bases in reads / 3.1 Gbp) giving a mean count of 48.15 ± 18.60 for HPRC samples. Except for bonobo, with a count of 40.24, no non-human primate had a copy count greater than 0.25 after normalizing for coverage. Lastly, we estimated the total number of rDNA units by concatenating two copies of a canonical rDNA repeat unit (KY962518) (Kim et al. 2018) and aligning all reads to it using MashMap v3.1.1 (Kille et al. 2023). The number of reads overlapping the ORF3 locus in the canonical sequence at over 99% identity was normalized by coverage to give an approximate rDNA copy number. This gave an average of 165.98 ± 49.48 in the HPRC samples. Based on this, we estimate 29.12 ± 7.77% of rDNA copies have an ORF at the ORF3 locus, consistent with the observations in CHM13 (43 of 219 or 19.63%). Due to the high identity threshold, only chimpanzee and bonobo rDNA counts could be estimated, with 354.39 and 188.58 copies, respectively.

We found that ORFs with one base change were approximately twice as frequent in the HPRC samples than those matching the TAR-cloned sequence (25% vs 13%). However, we also observed frame-preserving indels (43%) and ORFs with 2 or more substitutions (19%). We used MUSCLE v5.1 (Edgar 2004) with the command:

*muscle -super5 < input orf.fasta > -output orf.aln*

To build a core alignment for the 1-change ORFs, 2-change ORFs, and 2 + change ORFs, we filtered rare indels (likely due to sequencing errors in HiFi data) and visualized the alignments with Jalview (Waterhouse et al. 2009). Over 94% of the ORFs with one change have a G > C residue change at position 385 in nucleotide space. The remaining sequences match the C residue but have 1 difference elsewhere in the ORF, most commonly in homopolymer stretches and are likely sequencing errors due to their low frequency. The ORFs with 2 or more changes had a more complex structure, with the most common substitutions of G > C at 320, T > C at 374, C > G at 385, T > C at 389, T > G at 501, T > C at 511, and G > A at 549.

## Supplementary Information

Below is the link to the electronic supplementary material.

Supplementary file1 (TXT 24967 KB)

Supplementary file2 (TXT 9927 KB)

Supplementary file3 (TXT 8102 KB)

Supplementary file4 (XLSX 11 KB)

Supplementary file5 (PPTX 2571 KB)

Supplementary file6 (DOCX 57 KB)
