# Supplementary material for: Analysis of a Novel Human Protein, ORF3, Encoded by Spacer rDNA
Source: J Mol Evol. 2025 Sep 19;93(5):650–64. doi: 10.1007/s00239-025-10269-1 (PMC12579671; doi:10.1007/s00239-025-10269-1)
Supplement: Supplementary file 5 — Supplementary file5 (PPTX 2571 KB) [file 239_2025_10269_MOESM5_ESM.pptx]

## Slide 1
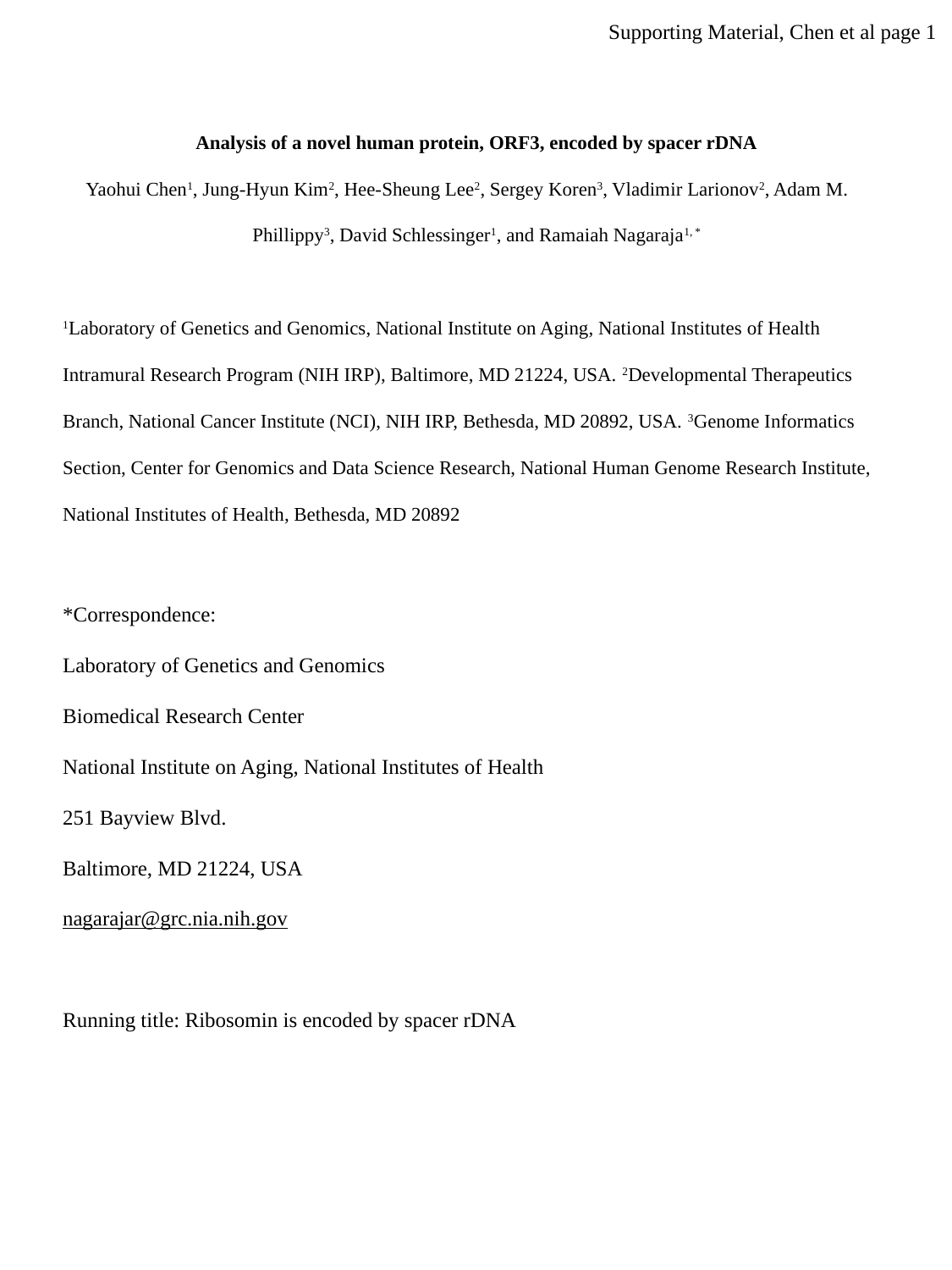

Supporting Material, Chen et al page 1
Analysis of a novel human protein, ORF3, encoded by spacer rDNA
Yaohui Chen1, Jung-Hyun Kim2, Hee-Sheung Lee2, Sergey Koren3, Vladimir Larionov2, Adam M. Phillippy3, David Schlessinger1, and Ramaiah Nagaraja1, *
1Laboratory of Genetics and Genomics, National Institute on Aging, National Institutes of Health Intramural Research Program (NIH IRP), Baltimore, MD 21224, USA. 2Developmental Therapeutics Branch, National Cancer Institute (NCI), NIH IRP, Bethesda, MD 20892, USA. 3Genome Informatics Section, Center for Genomics and Data Science Research, National Human Genome Research Institute, National Institutes of Health, Bethesda, MD 20892
*Correspondence:
Laboratory of Genetics and Genomics
Biomedical Research Center
National Institute on Aging, National Institutes of Health
251 Bayview Blvd.
Baltimore, MD 21224, USA
nagarajar@grc.nia.nih.gov
Running title: Ribosomin is encoded by spacer rDNA

## Slide 2
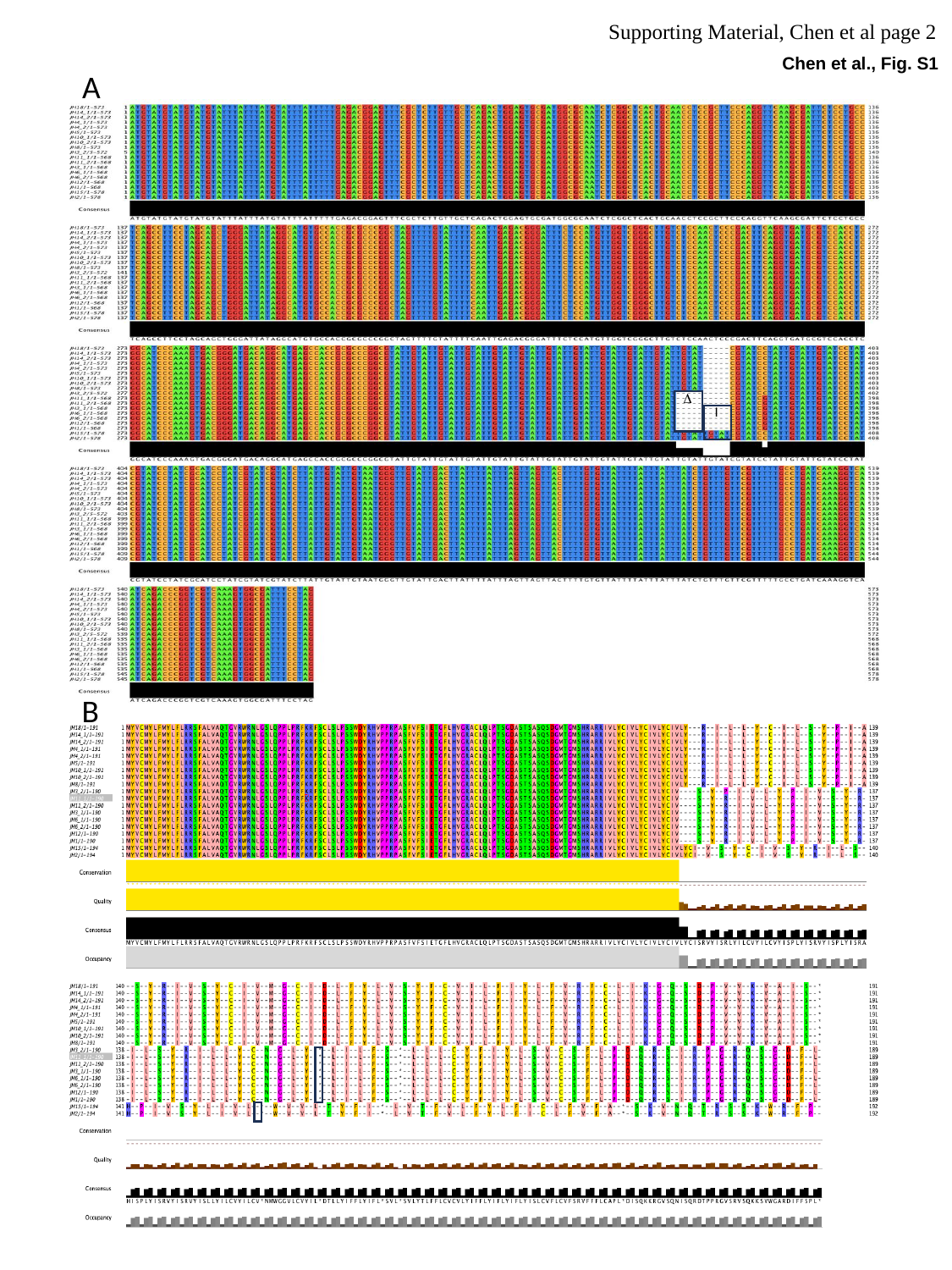

Supporting Material, Chen et al page 2
Chen et al., Fig. S1
A
D
I
Chen et al., Fig. S1
A
B
B

## Slide 3
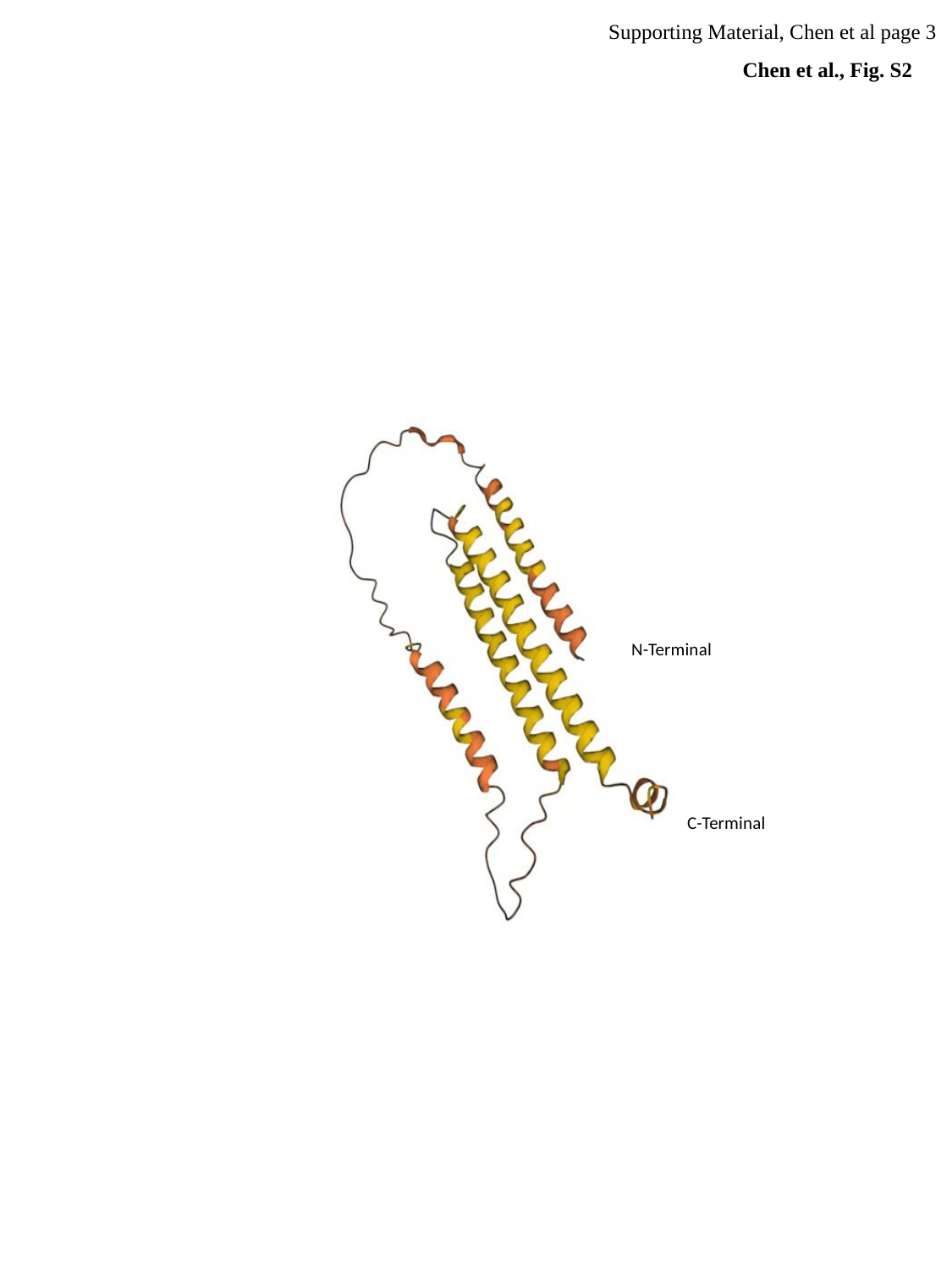

Supporting Material, Chen et al page 3
Chen et al., Fig. S2
N-Terminal
C-Terminal

## Slide 4
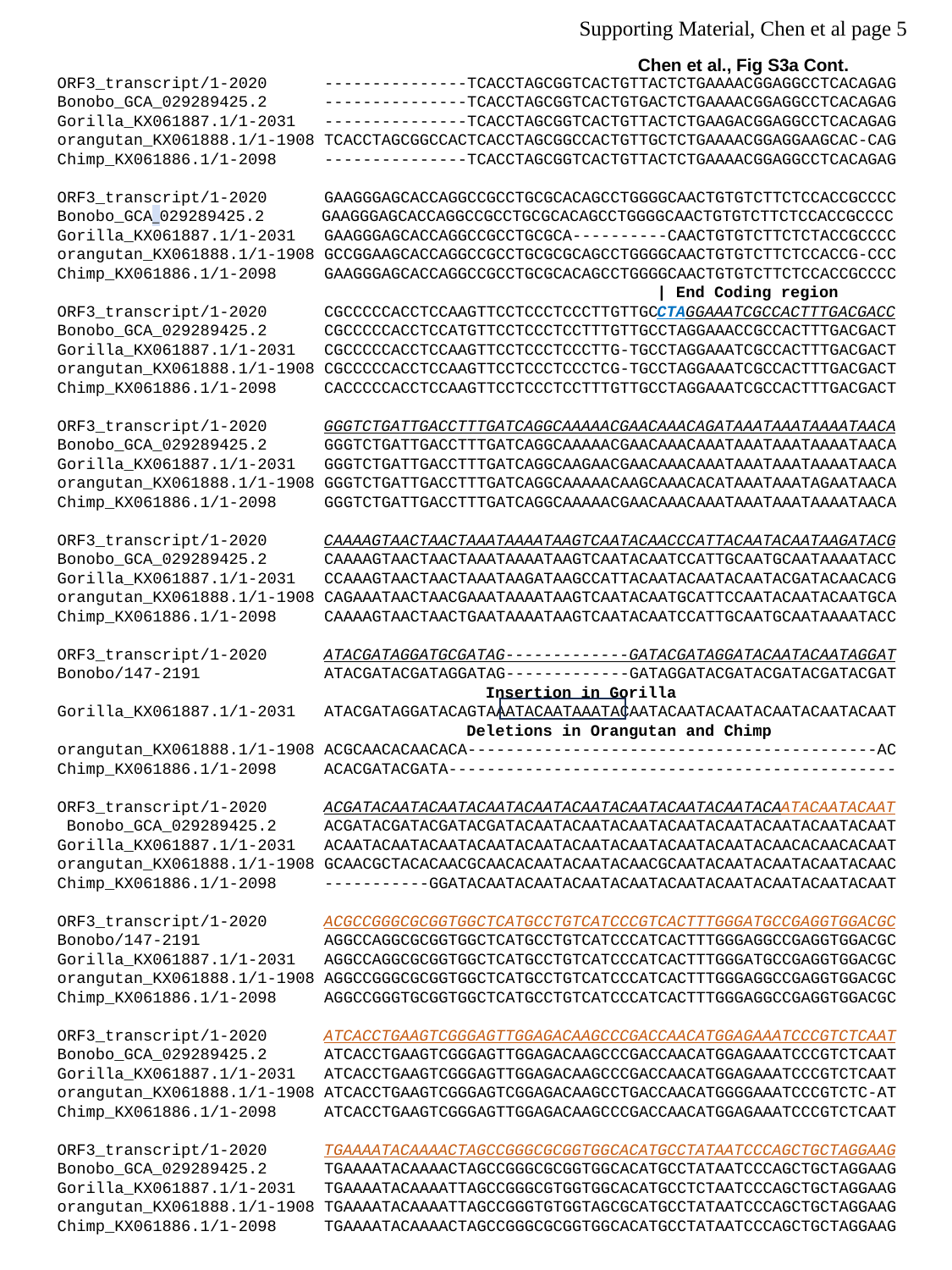

Supporting Material, Chen et al page 5
Chen et al., Fig S3a Cont.
ORF3_transcript/1-2020 ---------------TCACCTAGCGGTCACTGTTACTCTGAAAACGGAGGCCTCACAGAG
Bonobo_GCA_029289425.2 ---------------TCACCTAGCGGTCACTGTGACTCTGAAAACGGAGGCCTCACAGAG
Gorilla_KX061887.1/1-2031 ---------------TCACCTAGCGGTCACTGTTACTCTGAAGACGGAGGCCTCACAGAG
orangutan_KX061888.1/1-1908 TCACCTAGCGGCCACTCACCTAGCGGCCACTGTTGCTCTGAAAACGGAGGAAGCAC-CAG
Chimp_KX061886.1/1-2098 ---------------TCACCTAGCGGTCACTGTTACTCTGAAAACGGAGGCCTCACAGAG
ORF3_transcript/1-2020 GAAGGGAGCACCAGGCCGCCTGCGCACAGCCTGGGGCAACTGTGTCTTCTCCACCGCCCC
Bonobo_GCA_029289425.2 GAAGGGAGCACCAGGCCGCCTGCGCACAGCCTGGGGCAACTGTGTCTTCTCCACCGCCCC
Gorilla_KX061887.1/1-2031 GAAGGGAGCACCAGGCCGCCTGCGCA----------CAACTGTGTCTTCTCTACCGCCCC
orangutan_KX061888.1/1-1908 GCCGGAAGCACCAGGCCGCCTGCGCGCAGCCTGGGGCAACTGTGTCTTCTCCACCG-CCC
Chimp_KX061886.1/1-2098 GAAGGGAGCACCAGGCCGCCTGCGCACAGCCTGGGGCAACTGTGTCTTCTCCACCGCCCC
 | End Coding region
ORF3_transcript/1-2020 CGCCCCCACCTCCAAGTTCCTCCCTCCCTTGTTGCCTAGGAAATCGCCACTTTGACGACC
Bonobo_GCA_029289425.2 CGCCCCCACCTCCATGTTCCTCCCTCCTTTGTTGCCTAGGAAACCGCCACTTTGACGACT
Gorilla_KX061887.1/1-2031 CGCCCCCACCTCCAAGTTCCTCCCTCCCTTG-TGCCTAGGAAATCGCCACTTTGACGACT
orangutan_KX061888.1/1-1908 CGCCCCCACCTCCAAGTTCCTCCCTCCCTCG-TGCCTAGGAAATCGCCACTTTGACGACT
Chimp_KX061886.1/1-2098 CACCCCCACCTCCAAGTTCCTCCCTCCTTTGTTGCCTAGGAAATCGCCACTTTGACGACT
ORF3_transcript/1-2020 GGGTCTGATTGACCTTTGATCAGGCAAAAACGAACAAACAGATAAATAAATAAAATAACA
Bonobo_GCA_029289425.2 GGGTCTGATTGACCTTTGATCAGGCAAAAACGAACAAACAAATAAATAAATAAAATAACA
Gorilla_KX061887.1/1-2031 GGGTCTGATTGACCTTTGATCAGGCAAGAACGAACAAACAAATAAATAAATAAAATAACA
orangutan_KX061888.1/1-1908 GGGTCTGATTGACCTTTGATCAGGCAAAAACAAGCAAACACATAAATAAATAGAATAACA
Chimp_KX061886.1/1-2098 GGGTCTGATTGACCTTTGATCAGGCAAAAACGAACAAACAAATAAATAAATAAAATAACA
ORF3_transcript/1-2020 CAAAAGTAACTAACTAAATAAAATAAGTCAATACAACCCATTACAATACAATAAGATACG
Bonobo_GCA_029289425.2 CAAAAGTAACTAACTAAATAAAATAAGTCAATACAATCCATTGCAATGCAATAAAATACC
Gorilla_KX061887.1/1-2031 CCAAAGTAACTAACTAAATAAGATAAGCCATTACAATACAATACAATACGATACAACACG
orangutan_KX061888.1/1-1908 CAGAAATAACTAACGAAATAAAATAAGTCAATACAATGCATTCCAATACAATACAATGCA
Chimp_KX061886.1/1-2098 CAAAAGTAACTAACTGAATAAAATAAGTCAATACAATCCATTGCAATGCAATAAAATACC
ORF3_transcript/1-2020 ATACGATAGGATGCGATAG-------------GATACGATAGGATACAATACAATAGGAT
Bonobo/147-2191 ATACGATACGATAGGATAG-------------GATAGGATACGATACGATACGATACGAT
 Insertion in Gorilla
Gorilla_KX061887.1/1-2031 ATACGATAGGATACAGTAAATACAATAAATACAATACAATACAATACAATACAATACAAT
 Deletions in Orangutan and Chimp
orangutan_KX061888.1/1-1908 ACGCAACACAACACA-------------------------------------------AC
Chimp_KX061886.1/1-2098 ACACGATACGATA-----------------------------------------------
ORF3_transcript/1-2020 ACGATACAATACAATACAATACAATACAATACAATACAATACAATACAATACAATACAAT
 Bonobo_GCA_029289425.2 ACGATACGATACGATACGATACAATACAATACAATACAATACAATACAATACAATACAAT
Gorilla_KX061887.1/1-2031 ACAATACAATACAATACAATACAATACAATACAATACAATACAATACAACACAACACAAT
orangutan_KX061888.1/1-1908 GCAACGCTACACAACGCAACACAATACAATACAACGCAATACAATACAATACAATACAAC
Chimp_KX061886.1/1-2098 -----------GGATACAATACAATACAATACAATACAATACAATACAATACAATACAAT
ORF3_transcript/1-2020 ACGCCGGGCGCGGTGGCTCATGCCTGTCATCCCGTCACTTTGGGATGCCGAGGTGGACGC
Bonobo/147-2191 AGGCCAGGCGCGGTGGCTCATGCCTGTCATCCCATCACTTTGGGAGGCCGAGGTGGACGC
Gorilla_KX061887.1/1-2031 AGGCCAGGCGCGGTGGCTCATGCCTGTCATCCCATCACTTTGGGATGCCGAGGTGGACGC
orangutan_KX061888.1/1-1908 AGGCCGGGCGCGGTGGCTCATGCCTGTCATCCCATCACTTTGGGAGGCCGAGGTGGACGC
Chimp_KX061886.1/1-2098 AGGCCGGGTGCGGTGGCTCATGCCTGTCATCCCATCACTTTGGGAGGCCGAGGTGGACGC
ORF3_transcript/1-2020 ATCACCTGAAGTCGGGAGTTGGAGACAAGCCCGACCAACATGGAGAAATCCCGTCTCAAT
Bonobo_GCA_029289425.2 ATCACCTGAAGTCGGGAGTTGGAGACAAGCCCGACCAACATGGAGAAATCCCGTCTCAAT
Gorilla_KX061887.1/1-2031 ATCACCTGAAGTCGGGAGTTGGAGACAAGCCCGACCAACATGGAGAAATCCCGTCTCAAT
orangutan_KX061888.1/1-1908 ATCACCTGAAGTCGGGAGTCGGAGACAAGCCTGACCAACATGGGGAAATCCCGTCTC-AT
Chimp_KX061886.1/1-2098 ATCACCTGAAGTCGGGAGTTGGAGACAAGCCCGACCAACATGGAGAAATCCCGTCTCAAT
ORF3_transcript/1-2020 TGAAAATACAAAACTAGCCGGGCGCGGTGGCACATGCCTATAATCCCAGCTGCTAGGAAG
Bonobo_GCA_029289425.2 TGAAAATACAAAACTAGCCGGGCGCGGTGGCACATGCCTATAATCCCAGCTGCTAGGAAG
Gorilla_KX061887.1/1-2031 TGAAAATACAAAATTAGCCGGGCGTGGTGGCACATGCCTCTAATCCCAGCTGCTAGGAAG
orangutan_KX061888.1/1-1908 TGAAAATACAAAATTAGCCGGGTGTGGTAGCGCATGCCTATAATCCCAGCTGCTAGGAAG
Chimp_KX061886.1/1-2098 TGAAAATACAAAACTAGCCGGGCGCGGTGGCACATGCCTATAATCCCAGCTGCTAGGAAG

## Slide 5
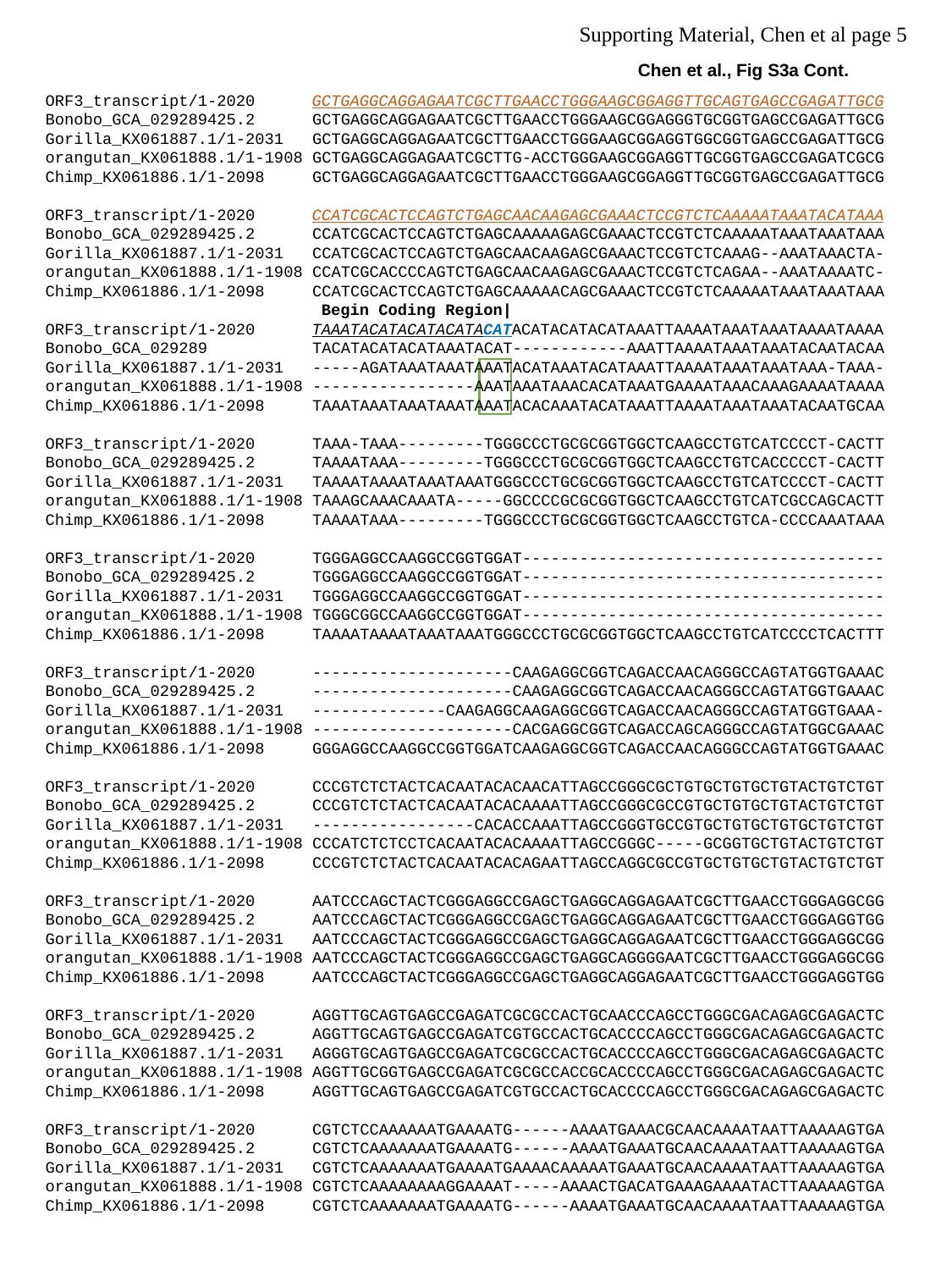

Supporting Material, Chen et al page 5
Chen et al., Fig S3a Cont.
ORF3_transcript/1-2020 GCTGAGGCAGGAGAATCGCTTGAACCTGGGAAGCGGAGGTTGCAGTGAGCCGAGATTGCG
Bonobo_GCA_029289425.2 GCTGAGGCAGGAGAATCGCTTGAACCTGGGAAGCGGAGGGTGCGGTGAGCCGAGATTGCG
Gorilla_KX061887.1/1-2031 GCTGAGGCAGGAGAATCGCTTGAACCTGGGAAGCGGAGGTGGCGGTGAGCCGAGATTGCG
orangutan_KX061888.1/1-1908 GCTGAGGCAGGAGAATCGCTTG-ACCTGGGAAGCGGAGGTTGCGGTGAGCCGAGATCGCG
Chimp_KX061886.1/1-2098 GCTGAGGCAGGAGAATCGCTTGAACCTGGGAAGCGGAGGTTGCGGTGAGCCGAGATTGCG
ORF3_transcript/1-2020 CCATCGCACTCCAGTCTGAGCAACAAGAGCGAAACTCCGTCTCAAAAATAAATACATAAA
Bonobo_GCA_029289425.2 CCATCGCACTCCAGTCTGAGCAAAAAGAGCGAAACTCCGTCTCAAAAATAAATAAATAAA
Gorilla_KX061887.1/1-2031 CCATCGCACTCCAGTCTGAGCAACAAGAGCGAAACTCCGTCTCAAAG--AAATAAACTA-
orangutan_KX061888.1/1-1908 CCATCGCACCCCAGTCTGAGCAACAAGAGCGAAACTCCGTCTCAGAA--AAATAAAATC-
Chimp_KX061886.1/1-2098 CCATCGCACTCCAGTCTGAGCAAAAACAGCGAAACTCCGTCTCAAAAATAAATAAATAAA
 Begin Coding Region|
ORF3_transcript/1-2020 TAAATACATACATACATACATACATACATACATAAATTAAAATAAATAAATAAAATAAAA
Bonobo_GCA_029289 TACATACATACATAAATACAT------------AAATTAAAATAAATAAATACAATACAA
Gorilla_KX061887.1/1-2031 -----AGATAAATAAATAAATACATAAATACATAAATTAAAATAAATAAATAAA-TAAA-
orangutan_KX061888.1/1-1908 -----------------AAATAAATAAACACATAAATGAAAATAAACAAAGAAAATAAAA
Chimp_KX061886.1/1-2098 TAAATAAATAAATAAATAAATACACAAATACATAAATTAAAATAAATAAATACAATGCAA
ORF3_transcript/1-2020 TAAA-TAAA---------TGGGCCCTGCGCGGTGGCTCAAGCCTGTCATCCCCT-CACTT
Bonobo_GCA_029289425.2 TAAAATAAA---------TGGGCCCTGCGCGGTGGCTCAAGCCTGTCACCCCCT-CACTT
Gorilla_KX061887.1/1-2031 TAAAATAAAATAAATAAATGGGCCCTGCGCGGTGGCTCAAGCCTGTCATCCCCT-CACTT
orangutan_KX061888.1/1-1908 TAAAGCAAACAAATA-----GGCCCCGCGCGGTGGCTCAAGCCTGTCATCGCCAGCACTT
Chimp_KX061886.1/1-2098 TAAAATAAA---------TGGGCCCTGCGCGGTGGCTCAAGCCTGTCA-CCCCAAATAAA
ORF3_transcript/1-2020 TGGGAGGCCAAGGCCGGTGGAT--------------------------------------
Bonobo_GCA_029289425.2 TGGGAGGCCAAGGCCGGTGGAT--------------------------------------
Gorilla_KX061887.1/1-2031 TGGGAGGCCAAGGCCGGTGGAT--------------------------------------
orangutan_KX061888.1/1-1908 TGGGCGGCCAAGGCCGGTGGAT--------------------------------------
Chimp_KX061886.1/1-2098 TAAAATAAAATAAATAAATGGGCCCTGCGCGGTGGCTCAAGCCTGTCATCCCCTCACTTT
ORF3_transcript/1-2020 ---------------------CAAGAGGCGGTCAGACCAACAGGGCCAGTATGGTGAAAC
Bonobo_GCA_029289425.2 ---------------------CAAGAGGCGGTCAGACCAACAGGGCCAGTATGGTGAAAC
Gorilla_KX061887.1/1-2031 --------------CAAGAGGCAAGAGGCGGTCAGACCAACAGGGCCAGTATGGTGAAA-
orangutan_KX061888.1/1-1908 ---------------------CACGAGGCGGTCAGACCAGCAGGGCCAGTATGGCGAAAC
Chimp_KX061886.1/1-2098 GGGAGGCCAAGGCCGGTGGATCAAGAGGCGGTCAGACCAACAGGGCCAGTATGGTGAAAC
ORF3_transcript/1-2020 CCCGTCTCTACTCACAATACACAACATTAGCCGGGCGCTGTGCTGTGCTGTACTGTCTGT
Bonobo_GCA_029289425.2 CCCGTCTCTACTCACAATACACAAAATTAGCCGGGCGCCGTGCTGTGCTGTACTGTCTGT
Gorilla_KX061887.1/1-2031 -----------------CACACCAAATTAGCCGGGTGCCGTGCTGTGCTGTGCTGTCTGT
orangutan_KX061888.1/1-1908 CCCATCTCTCCTCACAATACACAAAATTAGCCGGGC-----GCGGTGCTGTACTGTCTGT
Chimp_KX061886.1/1-2098 CCCGTCTCTACTCACAATACACAGAATTAGCCAGGCGCCGTGCTGTGCTGTACTGTCTGT
ORF3_transcript/1-2020 AATCCCAGCTACTCGGGAGGCCGAGCTGAGGCAGGAGAATCGCTTGAACCTGGGAGGCGG
Bonobo_GCA_029289425.2 AATCCCAGCTACTCGGGAGGCCGAGCTGAGGCAGGAGAATCGCTTGAACCTGGGAGGTGG
Gorilla_KX061887.1/1-2031 AATCCCAGCTACTCGGGAGGCCGAGCTGAGGCAGGAGAATCGCTTGAACCTGGGAGGCGG
orangutan_KX061888.1/1-1908 AATCCCAGCTACTCGGGAGGCCGAGCTGAGGCAGGGGAATCGCTTGAACCTGGGAGGCGG
Chimp_KX061886.1/1-2098 AATCCCAGCTACTCGGGAGGCCGAGCTGAGGCAGGAGAATCGCTTGAACCTGGGAGGTGG
ORF3_transcript/1-2020 AGGTTGCAGTGAGCCGAGATCGCGCCACTGCAACCCAGCCTGGGCGACAGAGCGAGACTC
Bonobo_GCA_029289425.2 AGGTTGCAGTGAGCCGAGATCGTGCCACTGCACCCCAGCCTGGGCGACAGAGCGAGACTC
Gorilla_KX061887.1/1-2031 AGGGTGCAGTGAGCCGAGATCGCGCCACTGCACCCCAGCCTGGGCGACAGAGCGAGACTC
orangutan_KX061888.1/1-1908 AGGTTGCGGTGAGCCGAGATCGCGCCACCGCACCCCAGCCTGGGCGACAGAGCGAGACTC
Chimp_KX061886.1/1-2098 AGGTTGCAGTGAGCCGAGATCGTGCCACTGCACCCCAGCCTGGGCGACAGAGCGAGACTC
ORF3_transcript/1-2020 CGTCTCCAAAAAATGAAAATG------AAAATGAAACGCAACAAAATAATTAAAAAGTGA
Bonobo_GCA_029289425.2 CGTCTCAAAAAAATGAAAATG------AAAATGAAATGCAACAAAATAATTAAAAAGTGA
Gorilla_KX061887.1/1-2031 CGTCTCAAAAAAATGAAAATGAAAACAAAAATGAAATGCAACAAAATAATTAAAAAGTGA
orangutan_KX061888.1/1-1908 CGTCTCAAAAAAAAGGAAAAT-----AAAACTGACATGAAAGAAAATACTTAAAAAGTGA
Chimp_KX061886.1/1-2098 CGTCTCAAAAAAATGAAAATG------AAAATGAAATGCAACAAAATAATTAAAAAGTGA

## Slide 6
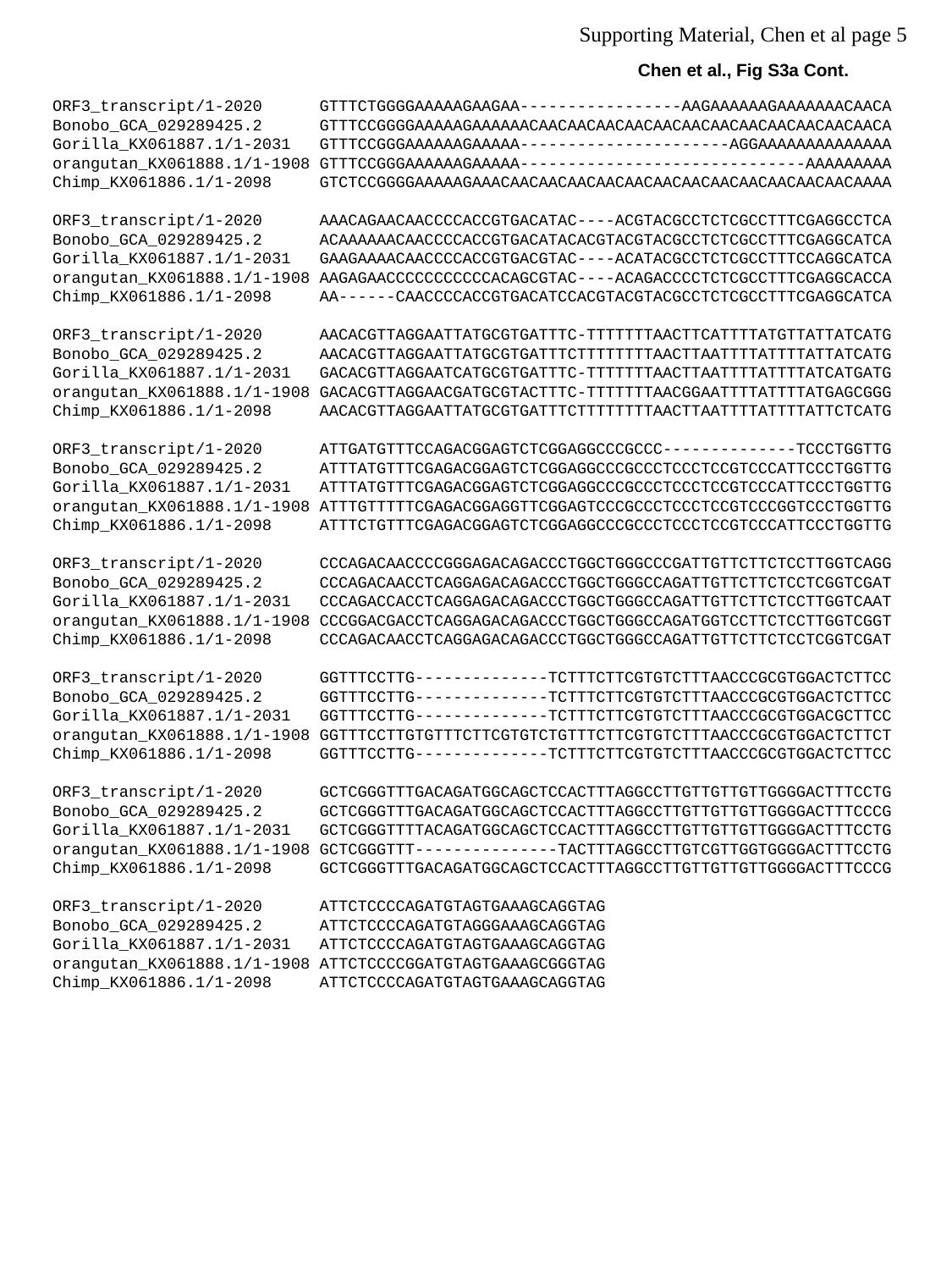

Supporting Material, Chen et al page 5
Chen et al., Fig S3a Cont.
ORF3_transcript/1-2020 GTTTCTGGGGAAAAAGAAGAA-----------------AAGAAAAAAGAAAAAAACAACA
Bonobo_GCA_029289425.2 GTTTCCGGGGAAAAAGAAAAAACAACAACAACAACAACAACAACAACAACAACAACAACA
Gorilla_KX061887.1/1-2031 GTTTCCGGGAAAAAAGAAAAA----------------------AGGAAAAAAAAAAAAAA
orangutan_KX061888.1/1-1908 GTTTCCGGGAAAAAAGAAAAA------------------------------AAAAAAAAA
Chimp_KX061886.1/1-2098 GTCTCCGGGGAAAAAGAAACAACAACAACAACAACAACAACAACAACAACAACAACAAAA
ORF3_transcript/1-2020 AAACAGAACAACCCCACCGTGACATAC----ACGTACGCCTCTCGCCTTTCGAGGCCTCA
Bonobo_GCA_029289425.2 ACAAAAAACAACCCCACCGTGACATACACGTACGTACGCCTCTCGCCTTTCGAGGCATCA
Gorilla_KX061887.1/1-2031 GAAGAAAACAACCCCACCGTGACGTAC----ACATACGCCTCTCGCCTTTCCAGGCATCA
orangutan_KX061888.1/1-1908 AAGAGAACCCCCCCCCCCACAGCGTAC----ACAGACCCCTCTCGCCTTTCGAGGCACCA
Chimp_KX061886.1/1-2098 AA------CAACCCCACCGTGACATCCACGTACGTACGCCTCTCGCCTTTCGAGGCATCA
ORF3_transcript/1-2020 AACACGTTAGGAATTATGCGTGATTTC-TTTTTTTAACTTCATTTTATGTTATTATCATG
Bonobo_GCA_029289425.2 AACACGTTAGGAATTATGCGTGATTTCTTTTTTTTAACTTAATTTTATTTTATTATCATG
Gorilla_KX061887.1/1-2031 GACACGTTAGGAATCATGCGTGATTTC-TTTTTTTAACTTAATTTTATTTTATCATGATG
orangutan_KX061888.1/1-1908 GACACGTTAGGAACGATGCGTACTTTC-TTTTTTTAACGGAATTTTATTTTATGAGCGGG
Chimp_KX061886.1/1-2098 AACACGTTAGGAATTATGCGTGATTTCTTTTTTTTAACTTAATTTTATTTTATTCTCATG
ORF3_transcript/1-2020 ATTGATGTTTCCAGACGGAGTCTCGGAGGCCCGCCC--------------TCCCTGGTTG
Bonobo_GCA_029289425.2 ATTTATGTTTCGAGACGGAGTCTCGGAGGCCCGCCCTCCCTCCGTCCCATTCCCTGGTTG
Gorilla_KX061887.1/1-2031 ATTTATGTTTCGAGACGGAGTCTCGGAGGCCCGCCCTCCCTCCGTCCCATTCCCTGGTTG
orangutan_KX061888.1/1-1908 ATTTGTTTTTCGAGACGGAGGTTCGGAGTCCCGCCCTCCCTCCGTCCCGGTCCCTGGTTG
Chimp_KX061886.1/1-2098 ATTTCTGTTTCGAGACGGAGTCTCGGAGGCCCGCCCTCCCTCCGTCCCATTCCCTGGTTG
ORF3_transcript/1-2020 CCCAGACAACCCCGGGAGACAGACCCTGGCTGGGCCCGATTGTTCTTCTCCTTGGTCAGG
Bonobo_GCA_029289425.2 CCCAGACAACCTCAGGAGACAGACCCTGGCTGGGCCAGATTGTTCTTCTCCTCGGTCGAT
Gorilla_KX061887.1/1-2031 CCCAGACCACCTCAGGAGACAGACCCTGGCTGGGCCAGATTGTTCTTCTCCTTGGTCAAT
orangutan_KX061888.1/1-1908 CCCGGACGACCTCAGGAGACAGACCCTGGCTGGGCCAGATGGTCCTTCTCCTTGGTCGGT
Chimp_KX061886.1/1-2098 CCCAGACAACCTCAGGAGACAGACCCTGGCTGGGCCAGATTGTTCTTCTCCTCGGTCGAT
ORF3_transcript/1-2020 GGTTTCCTTG--------------TCTTTCTTCGTGTCTTTAACCCGCGTGGACTCTTCC
Bonobo_GCA_029289425.2 GGTTTCCTTG--------------TCTTTCTTCGTGTCTTTAACCCGCGTGGACTCTTCC
Gorilla_KX061887.1/1-2031 GGTTTCCTTG--------------TCTTTCTTCGTGTCTTTAACCCGCGTGGACGCTTCC
orangutan_KX061888.1/1-1908 GGTTTCCTTGTGTTTCTTCGTGTCTGTTTCTTCGTGTCTTTAACCCGCGTGGACTCTTCT
Chimp_KX061886.1/1-2098 GGTTTCCTTG--------------TCTTTCTTCGTGTCTTTAACCCGCGTGGACTCTTCC
ORF3_transcript/1-2020 GCTCGGGTTTGACAGATGGCAGCTCCACTTTAGGCCTTGTTGTTGTTGGGGACTTTCCTG
Bonobo_GCA_029289425.2 GCTCGGGTTTGACAGATGGCAGCTCCACTTTAGGCCTTGTTGTTGTTGGGGACTTTCCCG
Gorilla_KX061887.1/1-2031 GCTCGGGTTTTACAGATGGCAGCTCCACTTTAGGCCTTGTTGTTGTTGGGGACTTTCCTG
orangutan_KX061888.1/1-1908 GCTCGGGTTT---------------TACTTTAGGCCTTGTCGTTGGTGGGGACTTTCCTG
Chimp_KX061886.1/1-2098 GCTCGGGTTTGACAGATGGCAGCTCCACTTTAGGCCTTGTTGTTGTTGGGGACTTTCCCG
ORF3_transcript/1-2020 ATTCTCCCCAGATGTAGTGAAAGCAGGTAG
Bonobo_GCA_029289425.2 ATTCTCCCCAGATGTAGGGAAAGCAGGTAG
Gorilla_KX061887.1/1-2031 ATTCTCCCCAGATGTAGTGAAAGCAGGTAG
orangutan_KX061888.1/1-1908 ATTCTCCCCGGATGTAGTGAAAGCGGGTAG
Chimp_KX061886.1/1-2098 ATTCTCCCCAGATGTAGTGAAAGCAGGTAG

## Slide 7
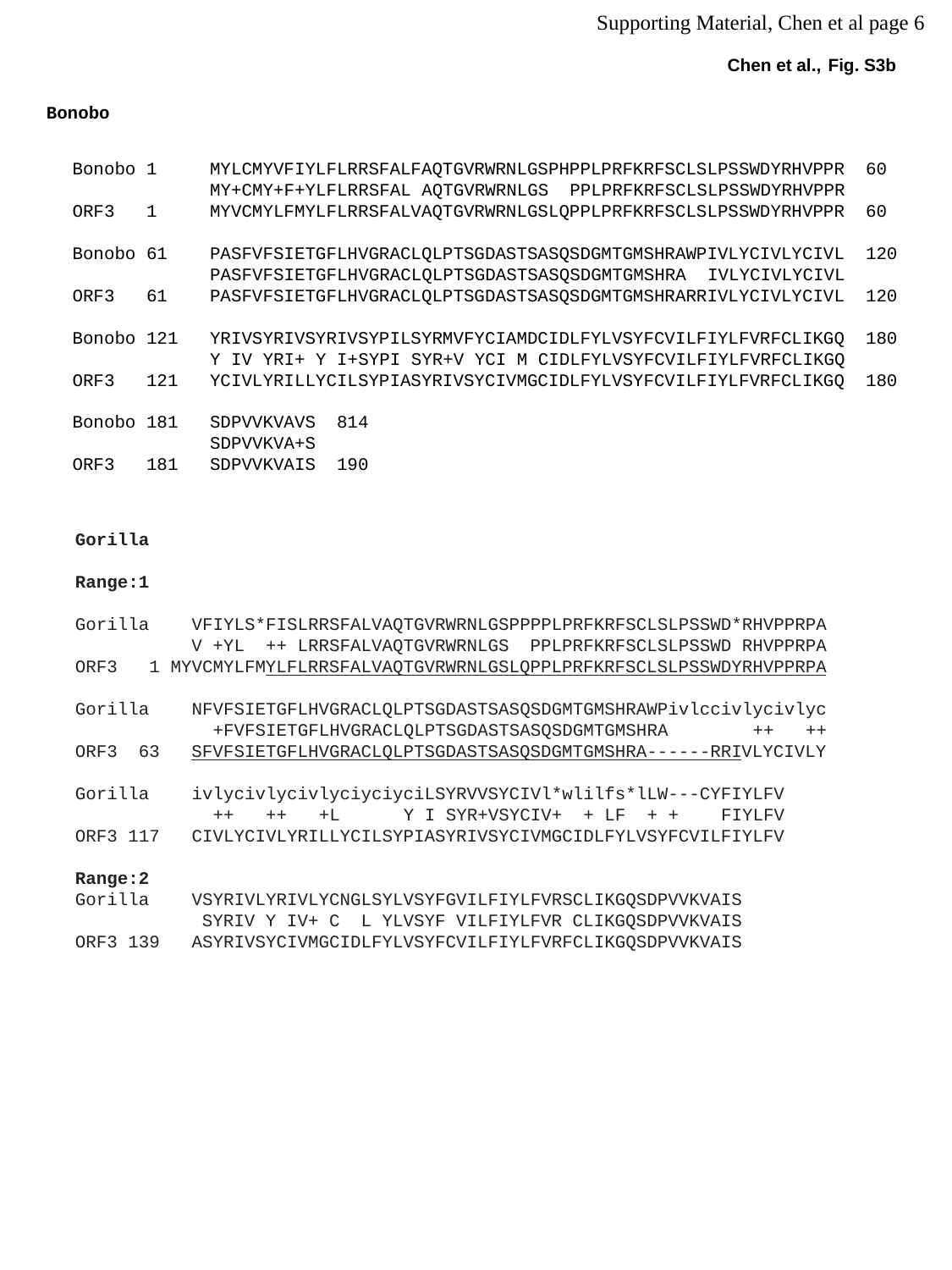

Supporting Material, Chen et al page 6
Chen et al., Fig. S3b
Bonobo
Bonobo 1 MYLCMYVFIYLFLRRSFALFAQTGVRWRNLGSPHPPLPRFKRFSCLSLPSSWDYRHVPPR 60
 MY+CMY+F+YLFLRRSFAL AQTGVRWRNLGS PPLPRFKRFSCLSLPSSWDYRHVPPR
ORF3 1 MYVCMYLFMYLFLRRSFALVAQTGVRWRNLGSLQPPLPRFKRFSCLSLPSSWDYRHVPPR 60
Bonobo 61 PASFVFSIETGFLHVGRACLQLPTSGDASTSASQSDGMTGMSHRAWPIVLYCIVLYCIVL 120
 PASFVFSIETGFLHVGRACLQLPTSGDASTSASQSDGMTGMSHRA IVLYCIVLYCIVL
ORF3 61 PASFVFSIETGFLHVGRACLQLPTSGDASTSASQSDGMTGMSHRARRIVLYCIVLYCIVL 120
Bonobo 121 YRIVSYRIVSYRIVSYPILSYRMVFYCIAMDCIDLFYLVSYFCVILFIYLFVRFCLIKGQ 180
 Y IV YRI+ Y I+SYPI SYR+V YCI M CIDLFYLVSYFCVILFIYLFVRFCLIKGQ
ORF3 121 YCIVLYRILLYCILSYPIASYRIVSYCIVMGCIDLFYLVSYFCVILFIYLFVRFCLIKGQ 180
Bonobo 181 SDPVVKVAVS 814
 SDPVVKVA+S
ORF3 181 SDPVVKVAIS 190
Gorilla
Range:1
Gorilla VFIYLS*FISLRRSFALVAQTGVRWRNLGSPPPPLPRFKRFSCLSLPSSWD*RHVPPRPA
 V +YL ++ LRRSFALVAQTGVRWRNLGS PPLPRFKRFSCLSLPSSWD RHVPPRPA
ORF3 1 MYVCMYLFMYLFLRRSFALVAQTGVRWRNLGSLQPPLPRFKRFSCLSLPSSWDYRHVPPRPA
Gorilla NFVFSIETGFLHVGRACLQLPTSGDASTSASQSDGMTGMSHRAWPivlccivlycivlyc
 +FVFSIETGFLHVGRACLQLPTSGDASTSASQSDGMTGMSHRA ++ ++
ORF3 63 SFVFSIETGFLHVGRACLQLPTSGDASTSASQSDGMTGMSHRA------RRIVLYCIVLY
Gorilla ivlycivlycivlyciyciyciLSYRVVSYCIVl*wlilfs*lLW---CYFIYLFV
 ++ ++ +L Y I SYR+VSYCIV+ + LF + + FIYLFV
ORF3 117 CIVLYCIVLYRILLYCILSYPIASYRIVSYCIVMGCIDLFYLVSYFCVILFIYLFV
Range:2
Gorilla VSYRIVLYRIVLYCNGLSYLVSYFGVILFIYLFVRSCLIKGQSDPVVKVAIS
 SYRIV Y IV+ C L YLVSYF VILFIYLFVR CLIKGQSDPVVKVAIS
ORF3 139 ASYRIVSYCIVMGCIDLFYLVSYFCVILFIYLFVRFCLIKGQSDPVVKVAIS

## Slide 8
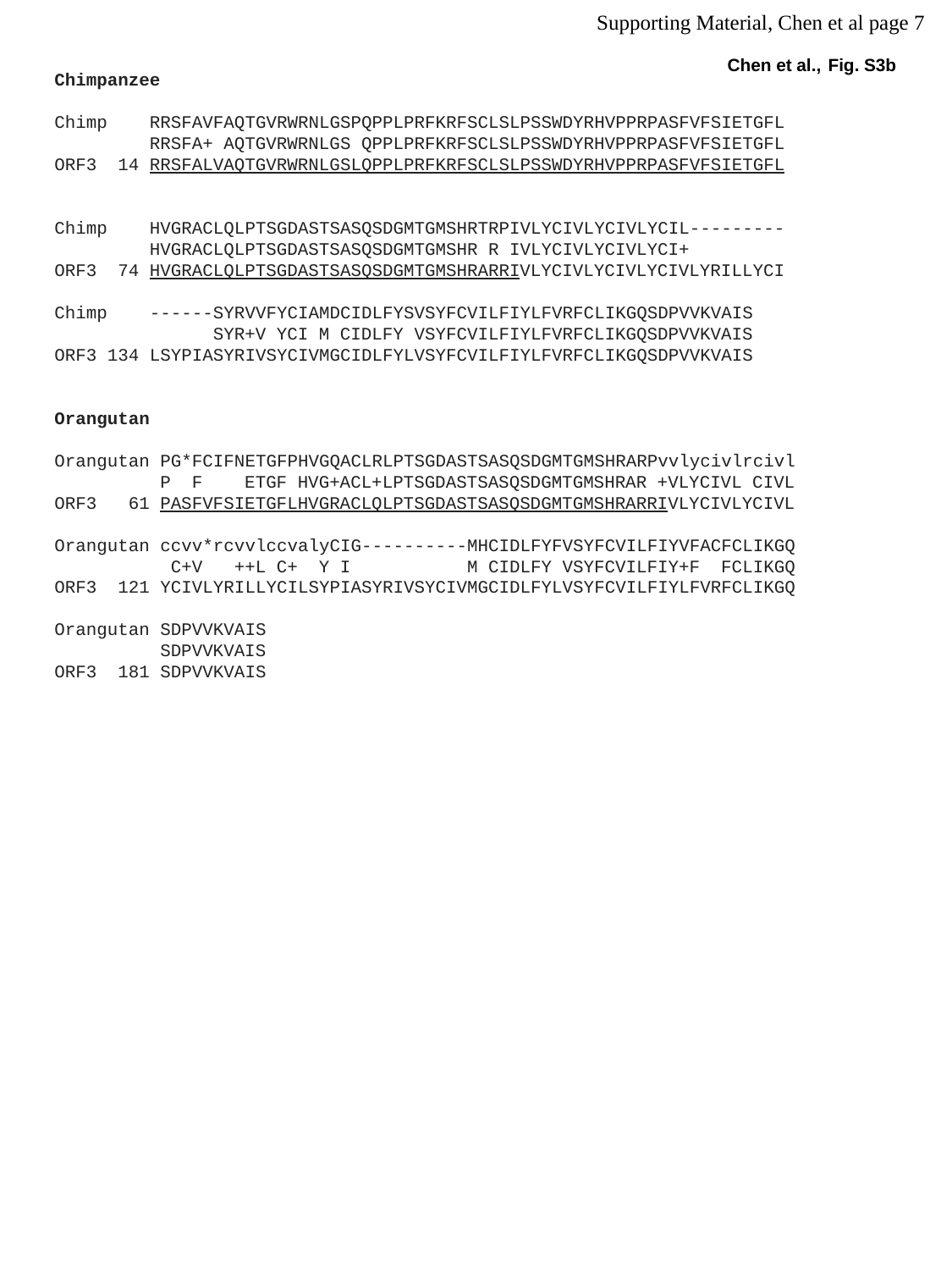

Supporting Material, Chen et al page 7
Chimpanzee
Chimp RRSFAVFAQTGVRWRNLGSPQPPLPRFKRFSCLSLPSSWDYRHVPPRPASFVFSIETGFL
 RRSFA+ AQTGVRWRNLGS QPPLPRFKRFSCLSLPSSWDYRHVPPRPASFVFSIETGFL
ORF3 14 RRSFALVAQTGVRWRNLGSLQPPLPRFKRFSCLSLPSSWDYRHVPPRPASFVFSIETGFL
Chimp HVGRACLQLPTSGDASTSASQSDGMTGMSHRTRPIVLYCIVLYCIVLYCIL---------
 HVGRACLQLPTSGDASTSASQSDGMTGMSHR R IVLYCIVLYCIVLYCI+
ORF3 74 HVGRACLQLPTSGDASTSASQSDGMTGMSHRARRIVLYCIVLYCIVLYCIVLYRILLYCI
Chimp ------SYRVVFYCIAMDCIDLFYSVSYFCVILFIYLFVRFCLIKGQSDPVVKVAIS
 SYR+V YCI M CIDLFY VSYFCVILFIYLFVRFCLIKGQSDPVVKVAIS
ORF3 134 LSYPIASYRIVSYCIVMGCIDLFYLVSYFCVILFIYLFVRFCLIKGQSDPVVKVAIS
Orangutan
Orangutan PG*FCIFNETGFPHVGQACLRLPTSGDASTSASQSDGMTGMSHRARPvvlycivlrcivl
 P F ETGF HVG+ACL+LPTSGDASTSASQSDGMTGMSHRAR +VLYCIVL CIVL
ORF3 61 PASFVFSIETGFLHVGRACLQLPTSGDASTSASQSDGMTGMSHRARRIVLYCIVLYCIVL
Orangutan ccvv*rcvvlccvalyCIG----------MHCIDLFYFVSYFCVILFIYVFACFCLIKGQ
 C+V ++L C+ Y I M CIDLFY VSYFCVILFIY+F FCLIKGQ
ORF3 121 YCIVLYRILLYCILSYPIASYRIVSYCIVMGCIDLFYLVSYFCVILFIYLFVRFCLIKGQ
Orangutan SDPVVKVAIS
 SDPVVKVAIS
ORF3 181 SDPVVKVAIS
Chen et al., Fig. S3b

## Slide 9
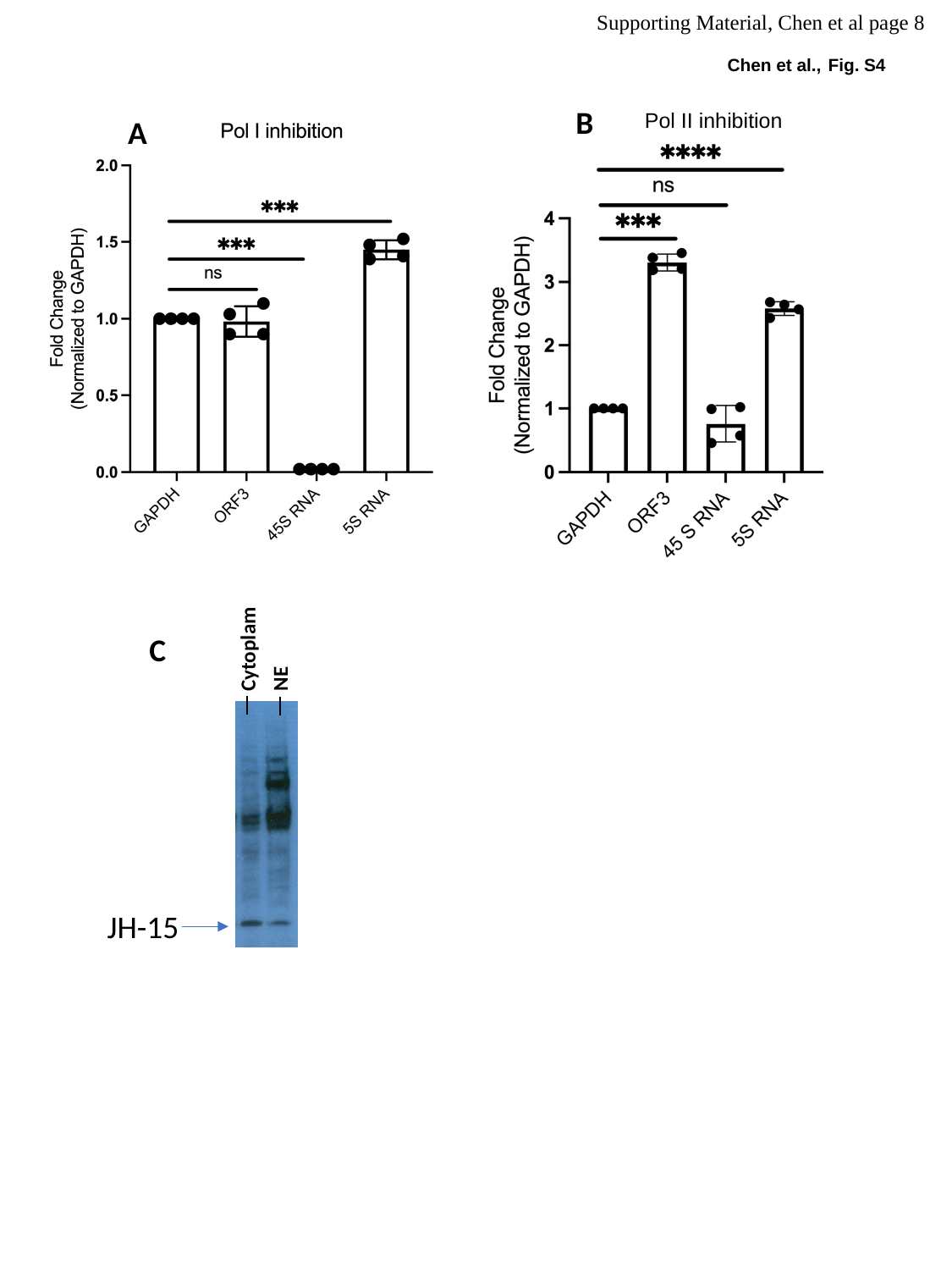

Supporting Material, Chen et al page 8
Chen et al., Fig. S4
B
Pol II inhibition
A
C
Cytoplam
NE
JH-15
