# Supplementary material for: Analysis of a Novel Human Protein, ORF3, Encoded by Spacer rDNA
Source: J Mol Evol. 2025 Sep 19;93(5):650–64. doi: 10.1007/s00239-025-10269-1 (PMC12579671; doi:10.1007/s00239-025-10269-1)
Supplement: Supplementary file 6 — Supplementary file6 (DOCX 57 KB) [file 239_2025_10269_MOESM6_ESM.docx]

**Supporting Information**

**S1 Fig. Nucleotide and protein alignments of *ORF3* coding sequence from Chromosome 21-specific BAC clones.**

(A) Nucleotide sequences for ORF3 in multiple BAC clones were aligned using the JalView program (Waterhouse et al. 2009). To facilitate pattern recognition, nucleotides are color-coded as A in green, T in blue, C in orange, and G in red. The image shows the complete ORF in 9 clones; 5 base deletions in 8 clones (Δ); and 5 base insertions (I) in 2 clones. (B) Coding sequence alignments from the same clones in panel (A) showing putative premature termination (boxed regions) due to deletions and insertions.

**S2 Fig. Alphafold structure prediction of ORF3.**

An mp4 file of Alphafold structure prediction showing possible helical regions of the protein with pLLD scores between <70 and >50 in yellow and <50 in red. Disordered regions are shown linking the predicted alpha helices. The N-terminal and C-terminal ends of the proteins are indicated.

**S3 Fig. Nucleotide and protein alignments with non-human primates.**

Nucleotide alignment of 2019 base human transcript with non-human primates; gorilla, chimpanzee, and orangutan showing conservation between them. A box highlights the absence of start codon in the non-human primates. The beginning and end of the ORF are marked [|] and the stretch of Alu insertion is highlighted in orange. B) Comparison of the ORF with non-human primates showing conserved stretches of ORF in each species.

S4 Fig. Representation of PolI and PolII inhibition and cellular fractionation.

1. Shows the plot following inhibition with BAMHI normalized to GAPDH. B) Shows plot following inhibition with DRB normalized to GAPDH. C) SDS polyacrylamide gel showing analysis of Cytoplasmic and Nuclear extracts and probed with anti-ORF antibody. The position of ORF is shown by an arrow head.

**S1 Table. List of primers used in this study.**

The sequences of primers used in this study are listed with a description of their purpose in the rows above the oligos. The Locked Nucleic Acid (LNA) primers used for inhibition experiments had 3 bases at the 5’ and 3’ ends with morpholino bases and are underlined.
